# Folate network genetic variation, plasma homocysteine, and global genomic methylation content: a genetic association study

**DOI:** 10.1186/1471-2350-12-150

**Published:** 2011-11-21

**Authors:** Susan M Wernimont, Andrew G Clark, Patrick J Stover, Martin T Wells, Augusto A Litonjua, Scott T Weiss, J Michael Gaziano, Katherine L Tucker, Andrea Baccarelli, Joel Schwartz, Valentina Bollati, Patricia A Cassano

**Affiliations:** 1 Division of Nutritional Sciences, Cornell University, Ithaca, NY, USA; 2 Department of Molecular Biology & Genetics, Cornell University, Ithaca, NY, USA; 3 Department of Biological Statistics & Computational Biology, Cornell, Ithaca, NY, USA; 4 Channing Laboratory, Brigham and Women's Hospital, and Harvard Medical School, Boston, MA, USA; 5 Veterans Administration (VA) Normative Aging Study, VA Boston Healthcare System, and Division of Aging, Brigham & Women's Hospital, Boston, MA, USA; 6 Department of Health Sciences, Northeastern University, Boston, MA, USA; 7 Departments of Environmental Health and Epidemiology, Harvard University, Boston, MA, USA; 8 Center of Molecular and Genetic Epidemiology, Department of Environmental and Occupational Health, Università degli Studi di Milano and IRCCS Fondazione Ca' Granda Ospedale Maggiore Policlinico, Milan, Italy; 9 209 Savage Hall, Division of Nutritional Sciences, Cornell University, Ithaca, NY, USA

## Abstract

**Background:**

Sequence variants in genes functioning in folate-mediated one-carbon metabolism are hypothesized to lead to changes in levels of homocysteine and DNA methylation, which, in turn, are associated with risk of cardiovascular disease.

**Methods:**

330 SNPs in 52 genes were studied in relation to plasma homocysteine and global genomic DNA methylation. SNPs were selected based on functional effects and gene coverage, and assays were completed on the Illumina Goldengate platform. Age-, smoking-, and nutrient-adjusted genotype--phenotype associations were estimated in regression models.

**Results:**

Using a nominal P ≤ 0.005 threshold for statistical significance, 20 SNPs were associated with plasma homocysteine, 8 with Alu methylation, and 1 with LINE-1 methylation. Using a more stringent false discovery rate threshold, SNPs in *FTCD*, *SLC19A1*, and *SLC19A3 *genes remained associated with plasma homocysteine. Gene by vitamin B-6 interactions were identified for both Alu and LINE-1 methylation, and epistatic interactions with the *MTHFR *rs1801133 SNP were identified for the plasma homocysteine phenotype. Pleiotropy involving the *MTHFD1L *and *SARDH *genes for both plasma homocysteine and Alu methylation phenotypes was identified.

**Conclusions:**

No single gene was associated with all three phenotypes, and the set of the most statistically significant SNPs predictive of homocysteine or Alu or LINE-1 methylation was unique to each phenotype. Genetic variation in folate-mediated one-carbon metabolism, other than the well-known effects of the *MTHFR *c.665C>T (known as c.677 C>T, rs1801133, p.Ala222Val), is predictive of cardiovascular disease biomarkers.

## Background

Folate and other B vitamins play key roles in biologic processes important to health, including DNA synthesis and the generation of cellular methylation potential. Folate status is influenced by both dietary intake and variation in genes encoding folate-related enzymes, and altered folate status due to nutritional or genetic perturbations is associated with adverse outcomes, including birth defects, cardiovascular disease (CVD), and cancer [1].

Elevated plasma homocysteine, a sulfur-containing amino acid by-product of folate metabolism, is a marker of disturbed folate-mediated one-carbon metabolism, and is associated with an increased risk of CVD [2-5]. Homocysteine levels are modulated by nutrition, particularly folate and vitamin B-12 [6], and by genetic variants, including a well-studied SNP in the methylenetetrahydrofolate reductase gene *MTHFR *c.665C>T (known as c.677 C>T, rs1801133, p.Ala222Val)[7].

The association of homocysteine with CVD is hypothesized to be mediated, in part, by changes in DNA methylation [8]. Folate-mediated one-carbon metabolism is linked to DNA methylation status through regulation of S-adenosylmethionine, the universal methyl donor, and through the activity of enzymes involved in methylation reactions [9,10].

LINE-1 and Alu elements are abundant, transposable elements whose methylation status has been shown to be highly correlated with genome-wide DNA methylation in some studies [11,12]. Atherosclerosis is characterized by global DNA hypomethylation and transposable element methylation levels are associated with heart disease, stroke, and total mortality; reduced LINE-1 methylation was associated with an increased incidence of ischemic heart disease and stroke in the Normative Aging Study (NAS) [13]. These findings contribute to interest in global genomic DNA methylation as a potential biomarker of CVD risk.

Most previous work investigating variation in genes contributing to folate-mediated one-carbon metabolism in relation to homocysteine and genomic methylation phenotypes focused on a small number of candidate genes; however, other enzymes and genes may also be important; thus this study represents both first report and replication efforts. To investigate the genetic and nutritional predictors of homocysteine and methylation phenotypes, this candidate gene study examined variation across the network of genes representing folate-mediated one-carbon metabolism in relation to homocysteine and methylation outcomes. 330 single nucleotide polymorphisms (SNPs) in 52 genes with a role in folate-mediated one-carbon metabolism were studied. The set of genes, the SNP markers, and the nutrients examined in this study were selected to represent the full functional variation of the folate-mediated one carbon metabolic pathway.

## Methods

### Study population

The Veterans' Administration (VA) established the NAS in 1961. 2,280 men aged 21-81 years (mean age of 42 y at study entry) were enrolled in the study on the basis of health criteria; details have been described elsewhere [14,15]. The analyses described herein focus on non-Hispanic white males using data from the subset of men (~ 700) with measurements of homocysteine and global genomic DNA methylation (Alu and LINE-1). This study complied with the Helsinki Declaration and was approved by the following: Brigham and Women's Hospital Human Subjects committee, VA R&D committee, Harvard School of Public Health, Cornell University Committee on Human Subjects.

### DNA extraction, SNP selection and genotyping

Genomic DNA was extracted from stored frozen buffy coat of 7 ml whole blood using the QIAamp DNA Blood Kit (QIAGEN, Valencia, CA). The REPLI-g whole genome amplification kit (QIAGEN) was used to amplify genomic DNA when quantity was insufficient for genotyping.

52 genes that contribute to folate-mediated one-carbon metabolism were identified (Additional file 1). SNP selection encompassed 2 kb on either side of the gene to include promoter and/or regulatory region variants; a total of 384 SNPs were selected. 384 SNPs were submitted to the Center for Inherited Disease Research at the Johns Hopkins University for genotyping via an Illumina GoldenGate custom genotyping panel. Genotype frequencies in controls were compared with those expected in Hardy-Weinberg equilibrium (HWE). Of the 384 SNPs originally submitted, 54 were ultimately excluded, leaving 330 SNPs available for analysis (Additional file 2).

Extensive previously collected data on study participants includes physical measurements, lifestyle factors, and blood assays. Plasma folate, vitamin B-6 (as pyridoxal-5'-phosphate; PLP) and vitamin B-12 were assayed as previously described [16]. Plasma total homocysteine was assayed in the same unselected subset of stored blood samples as plasma folate, vitamin B-6, and vitamin B-12 [16]. The analysis of transposon DNA methylation was reported in prior publications [17,18].

Restricted maximum likelihood and ordinary least squares regression models evaluated the relation between SNPs and the plasma homocysteine and global DNA methylation phenotypes; maximum likelihood regression was used to evaluate epistatic interactions with the dummy-coded *MTHFR *SNP. Previous work in this cohort demonstrated no population substructure [19], thus no adjustments were made. All regression models were adjusted for age, smoking status, and nutrient residuals (variation in nutrient not predicted by SNP), and an extended model also adjusted for the *MTHFR *rs1801133 variant (coded as recessive to account for the pattern of association using the fewest model terms). For the homocysteine phenotype, further models tested the interaction of each genotype with the rs1801133 SNP. For all phenotypes, further models tested the interaction of each genotype with the nutrients.

For main effects, regression coefficients with a nominal P ≤ 0.005 were reported, and a False Discovery Rate (FDR) multiple testing correction [20] was applied, with an FDR-adjusted P value significance threshold of 0.05; final models were conditional on a first step that selected the best genetic model for each SNP, thus the FDR is conditional on this first step. For interactions, a less stringent FDR-adjusted P value significance threshold of 0.20 was used. For gene-nutrient interactions, regression coefficients with a nominal P ≤ 0.02 were reported, given few results reached the FDR threshold.

To assess effect modification, product terms between the SNP and the nutrient biomarker residual were included in models. Interactions were captured in a single model term; significance of the interaction was assessed by the P value for the interaction term. Interactions with *MTHFR *rs1801133, which was dummy-coded, were assessed with the likelihood ratio test (LRT). All statistical analyses were conducted with SAS v. 9.2 (SAS, Cary, NC).

Additional details on methodology are provided in online materials (Additional file 3).

## Results

Measurements of the homocysteine phenotype, the Alu element methylation phenotype, and the LINE-1 methylation phenotype were available for 760, 628 and 621 participants, respectively. All had genotype data, 533 men had data on all three phenotypes; each analysis included the maximum number possible. The phenotype groups had similar frequencies for the *MTHFR *rs1801133 *TT *genotype, but differed by age and hence differed slightly on age-related variables (Table 1). The *MTHFR *rs1801133 *TT *genotype prevalence in the largest group, the plasma homocysteine group, was 12.2%, similar to the frequency reported in a large North American sample [7].

**Table 1:** Characteristics of Normative Aging Study participants, 1961-2001, with measurements on three phenotypes.

	**Plasma homocysteine**^**a**^	**Global genomic DNA methylation (Alu elements)**^**b**^	**Global genomic DNA methylation (LINE-1 elements)**^**b**^
	
	N = 760	N = 628	N = 621
Age at phenotype measurement	68.6 (7.3)*	72.5 (6.8)	72.5 (6.8)
Education - college graduate or higher (%)	26.8	28.6	28.7
White (%)	100	100	100
Baseline BMI (kg/m^2^)	25.9 (2.9)	25.9 (2.9)	25.9 (2.9)
Cigarette smoking^c^			
Current (%)	6.7	5.4	5.5
Former (%)	63.0	63.4	63.5
Never (%)	30.3	31.2	31.1
Alcohol intake (% consuming ≥ 2 drinks/day)	12.4	13.7	13.5
Baseline diabetes diagnosis (%)	0.13	0.16	0.16
Baseline systolic blood pressure (mm Hg)	122.1 (12.7)	121.7 (12.5)	121.6 (12.6)
*MTHFR 677 C>T *(rs1801133) *TT *genotype (%)	12.2	12.3	12.6
Plasma folate (ng/ml)^d^	10.4 (5.7)	17.2 (15.1)	17.0 (14.9)
Plasma vitamin B-6 (pmol/ml)	84.9 (85.3)	104.6 (96.1)	104.8 (96.7)
Plasma vitamin B-12 (pg/ml)	458.9 (190.6)	512.8 (371.2)	514.9 (373.8)
Plasma total homocysteine (nmol/ml)	10.6 (3.7)	11.0 (4.2)	10.9 (4.2)
Global DNA methylation in Alu elements (%)		26.3 (1.1)	
Global DNA methylation in LINE elements (%)			76.9 (1.9)

*mean (standard deviation) unless otherwise indicated

^a^N for homocysteine group ranges from 730 to 760 for variables in table.

^b^Men in the two global genomic methylation groups were very similar, and dates of marker collection were nearly identical. N for Alu group ranges from 618 to 628, and N for LINE-1 group ranges from 611 to 621.

^c^Smoking status was assessed using most recent data prior to phenotype measurement.

^d^Homocysteine and corresponding plasma folate, vitamin B-6, and vitamin B-12 data were collected *prior *to the initiation of folate fortification; global genomic methylation markers, and corresponding plasma nutrient markers were measured *post*-folate fortification, in a later time period.

**Figure 1:**
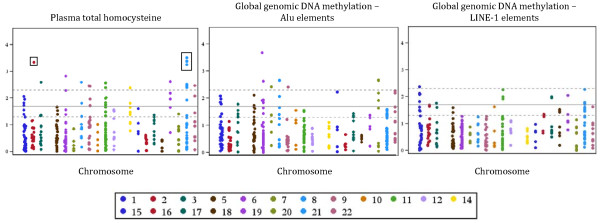
**Manhattan plot**. Folate-related SNPs as predictors of plasma total homocysteine and global genomic DNA methylation phenotypes. Models adjusted for age, smoking status, and folate, vitamin B-6, and vitamin B-12 residuals. Horizontal lines represent nominal P values of 0.05 (lower dashed line), 0.02 (center solid line) and 0.005 (upper dashed line). Boxes indicate SNPs that reached False Discovery Rate significance.

Age and current smoking status were associated with homocysteine (P ≤ 0.001), age was associated with Alu (P ≤ 0.005), and current smoking was associated with LINE-1 (P = 0.055). Folate, vitamin B-6, and vitamin B-12 were associated with homocysteine (P ≤ 0.005), vitamin B-6 was associated with Alu (P ≤ 0.05), and these biomarkers had little or no association with LINE-1. Models exploring the SNP--phenotype association were adjusted for age, smoking, and nutrient residuals. Adjusting for age and smoking made little difference to the coefficients for each SNP. The set of SNPs comprising the most significant associations was nearly identical with or without adjusting for nutrient residuals. Further adjustment for the *MTHFR *rs1801133 variant made little or no difference to the SNP regression coefficients. The most statistically significant SNPs for each phenotype were relatively common (MAF ≥13%), and the set of most significant SNPs was unique to each phenotype (Tables 2, 3, and 4 and Figure 1).

**Table 2:** The most statistically significant associations (P ≤ 0.005) between single nucleotide polymorphisms and the plasma homocysteine phenotype ^a, e^

Gene Name	rs#	Nominal P	**Effect**^**c**^	**Chr**.	Coded allele	Coded allele frequency	**Genetic Model**^**g**^	**SNP Type**^**h**^
*FTCD*	rs2277820	3.09E-04**^b^**	7.22%	21	*T*	26%	O	I
*SLC19A1*	rs1051266	4.16E-04**^b^**	5.04%	21	*A*	44%	A	CN
*SLC19A1*	rs1131596	4.31E-04**^b^**	5.03%	21	*C*	44%	A	5'
*SLC19A3*	rs13007334	4.61E-04**^b^**	6.89%	2	*C*	46%	O	I
*SLC19A1*	rs4819130	5.65E-04**^b^**	4.94%	21	*C*	44%	A	I
*MTHFD1L*	rs11754661^d^	1.51E-03	49.98%	6	*A*	7%	R	I
*DNMT1*	rs2228611	2.42E-03	-6.44%	19	*G*	49%	D	CS
*ALDH1L1*	rs3772424	2.52E-03	6.14%	3	*A*	20%	D	I
*GGH*	rs4617146	2.55E-03	5.31%	8	*T*	19%	A	I
*CELF1*	rs4752843	2.74E-03	-5.72%	11	*C*	14%	A	I
*SLC19A1*	rs12482346	3.02E-03	4.08%	21	*T*	44%	A	I
*SLC19A1*	rs2297291	3.39E-03	6.07%	21	*A*	41%	D	I
*TCN2*	rs4820886	3.41E-03	-17.30%	22	*G*	13%	R	I
*TCN2*	rs9621049	3.41E-03	-17.30%	22	*T*	13%	R	CN
*GLDC*	rs7848919	3.52E-03	5.70%	9	*G*	32%	D	3'
*SARDH*	rs2502741^f^	3.60E-03	6.60%	9	*A*	50%	D	I
*SLC19A1*	rs1051298	3.68E-03	3.98%	21	*T*	44%	A	3'
*CBS*	rs6586282	4.06E-03	-5.80%	21	*T*	18%	O	I
*FOLH1*	rs202673	4.08E-03	-19.30%	11	*G*	14%	R	I
*MTHFD1*	rs1950902^f^	4.19E-03	-5.18%	14	*T*	16%	A	CN

^a^Model adjusted for age, smoking, and residuals of plasma folate, plasma vitamin B-6, and plasma vitamin B-12; forward strand allele shown.

^b^FDR-adjusted P values reached False Discovery Rate significance threshold of 0.05.

^c^Effect is shown as percent change in plasma homocysteine levels.

^d^Sparse data (fewer than 5 individuals per category) for some genotype categories.

^e^No SNPs map to more than one gene.

^f^Lower quality SNP.

^g^D:Dominant; R:Recessive; A:Additive; O:Overdominant.

^h^5':5' region; 3':3' region; CN:Coding nonsynonymous; CS:Coding synonymous; I:Intronic.

**Table 3:** The most statistically significant associations (P ≤ 0.005) between single nucleotide polymorphisms and the Alu methylation phenotype a, b, d, f

Gene	rs#	Nominal P	**Effect**^**c**^	Chr	Coded allele	Coded allele frequency	**Genetic Model**^**g**^	**Type**^**h**^
*GNMT*	rs1051218^e^	2.14E-04	-0.57	6	*T*	3%	D	3'
*DNMT3B*	rs2424914	2.16E-03	0.30	20	*G*	45%	R	I
*SLC25A32*	rs3134297^e^	2.20E-03	0.65	8	*C*	20%	R	5'
*DNMT3B*	rs2424922	2.21E-03	0.30	20	*C*	45%	R	CS
*DNMT3B*	rs6058891	2.21E-03	0.30	20	*C*	45%	R	CS
*MTHFD1L*	rs1738574	2.39E-03	0.24	6	*T*	45%	O	I
*AHCYL2*	rs1665105	3.87E-03	0.16	7	*T*	44%	A	3'
*SARDH*	rs129886	3.92E-03	-0.60	9	*T*	19%	R	3'

^a^Model adjusted for age, smoking, and residuals of plasma folate, plasma vitamin B-6, and plasma vitamin B-12; forward strand allele shown;

^b^No FDR-adjusted P values reached False Discovery Rate significance threshold of 0.05.

^c^Effect represents per genotype change in Alu element methylation standard deviation.

^d^No sparse data (fewer than 5 individuals per category) for any genotype categories of these SNPs.

^e^SNP maps to more than one gene (rs1051218 also maps to *PEX6*, rs3134297 also maps to *WDSOF1/DCAF13*).

^f^No lower quality SNPs.

^g^D:Dominant; R:Recessive; A:Additive; O:Overdominant.

^h^5':5' region; 3':3' region; CN:Coding nonsynonymous; CS:Coding synonymous; I:Intronic.

**Table 4:** The most statistically significant association (P ≤ 0.005) between single nucleotide polymorphisms and the LINE-1 methylation phenotype ^a, b, d, e, f^

Gene	rs#	Nominal P	**Effect**^**c**^	Chr	Coded allele	Coded allele frequency	**Genetic Model**^**g**^	**Type**^**h**^
*MTHFR*	rs12121543	4.29E-03	0.48	1	*A*	24%	R	I

^a^Model adjusted for age, smoking, and residuals of plasma folate, plasma vitamin B-6, and plasma vitamin B-12; forward strand allele shown.

^b^No FDR-adjusted P values reached False Discovery Rate significance threshold of 0.05.

^c^Effect represents per genotype change in LINE-1 element methylation standard deviation.

^d^No sparse data (fewer than 5 individuals per category) for any genotype categories of these SNPs.

^e^No SNPs map to more than one gene.

^f^No lower quality SNPs.

^g^D:Dominant; R:Recessive; A:Additive; O:Overdominant.

^h^5':5' region; 3':3' region; CN:Coding nonsynonymous; CS:Coding synonymous; I:Intronic.

### Total plasma homocysteine phenotype

Of the 20 SNPs with a nominal P ≤ 0.005, five were also significant at the FDR threshold (P ≤ 0.05) (Table 2). These 5 SNPs comprise 3 genes: formiminotransferase cyclodeaminase (*FTCD*; 1 SNP, intronic), solute carrier family 19 (folate transporter), member 1 (*SLC19A1*, 3 SNPs, representing coding nonsynonymous, 5' region, and intronic variants), and solute carrier family 19, member 3 (*SLC19A3*, 1 SNP, intronic). Genetic variation in all 5 SNPs was positively associated with plasma homocysteine levels, and effects were similar in direction and magnitude (variant genotypes associated with a 4.9-7.2% higher plasma total homocysteine vs. the referent genotype). In each case, the association of the genotype with homocysteine was partially mediated by nutrients; when plasma folate and vitamin B-6 or B-12 biomarkers were added to the models, the regression coefficients were reduced by 29% for *FTCD *rs2277820, by 43% for *SLC19A1 *rs1051266, rs1131596, and rs4819130, and by 34% for *SLC19A3 *rs13007334 (data not shown). A model containing a nonredundant set of 3 of the top 5 FDR-significant SNPs (*FTCD *rs2277820, *SLC19A3 *rs13007334, *SLC19A1 *rs1051266) explained 3.6% of the variation in plasma homocysteine beyond that explained by age, smoking, and folate, B-6, and B-12 residuals (data not shown); the set of 3 SNPs was statistically significant (LRT = 17.6, P = 0.0005, 3 degrees of freedom, df), and the coefficients for each SNP were similar to coefficients from single SNP models. Considering the *MTHFR *genotype in more detail, the *TT *genotype group (vs. *CC*) had elevated homocysteine (nominal P = 0.0052), but the *CT *genotype had no association with homocysteine (nominal P = 0.8107); thus, the *MTHFR *genotype did not pass preset FDR thresholds.

In models investigating interactions between each SNP and *MTHFR *rs1801133, 4 interaction terms were below the FDR threshold (FDR-adjusted P value ≤ 0.2) for the homocysteine phenotype (Additional file 4). No SNP--nutrient (folate, B-6, or B-12) interaction coefficients reached FDR-significance (FDR-adjusted P value ≤ 0.2; Additional file 5). The *MTHFR*--folate interaction did not reach preset statistical thresholds (p_nominal _= 0.0578), but the pattern of interaction supported a greater association of *MTHFR TT *genotype with homocysteine conditional on lower folate status.

### Global genomic DNA methylation phenotype: Alu elements

In analyses of the Alu element methylation phenotype, 8 SNPs were statistically significant with a nominal P ≤ 0.005; however, none were statistically significant at the FDR threshold (FDR-adjusted P value ≤ 0.05) (Table 3). There was little or no mediation of the association by nutrients or plasma homocysteine levels (data not shown). There were no SNP--nutrient interactions with folate or B-12 that reached FDR thresholds for statistical significance (FDR-adjusted P ≤ 0.2) (Additional file 6). Three SNPs had an FDR-significant interaction with plasma vitamin B-6 (Additional file 6); these interactions involved 3 intronic SNPs in 2 genes, aminomethyltransferase (*AMT*, rs1464567 and rs1464566) and DNA (cytosine-5-)-methyltransferase 3 beta (*DNMT3B*, rs1883729). Comparing men with the *AMT *rs1464567 *CC/CG *genotype to the *GG *genotype, the mean Alu element methylation was 0.4 SD higher at low B-6, 0.1 SD higher at median B-6, and 0.4 SD lower at high B-6. Comparing men with the *AMT *rs1464566 *GG/GA *genotype to the *AA *genotype, the mean Alu element methylation was 0.4 SD higher at low B-6, 0.1 SD higher at median B-6, and 0.3 SD lower at high B-6. Comparing men with the *DNMT3B *rs1883729 *AA *genotype to the *AG/GG *genotype, the mean Alu element methylation was 0.1 SD lower at low B-6, 0.3 SD higher at median B-6, and 0.8 SD higher at high B-6.

### Global genomic DNA methylation phenotype: LINE-1 elements

No SNP main effect associations reached the FDR-significance threshold for LINE-1 methylation (FDR-adjusted P ≤ 0.05; Table 4). There were no SNP--nutrient interactions for folate or B-12 that reached FDR-significance levels (FDR-adjusted P ≤ 0.2) (Additional file 7). An interaction of plasma B-6 with 1 SNP was significant at the FDR threshold of P ≤ 0.2 (rs17080689, an intronic SNP in methylenetetrahydrofolate dehydrogenase (NADP+ dependent) 1-like, *MTHFD1L*) (Additional file 7), suggesting that the relation of the SNP to LINE-1 methylation varied according to plasma levels of vitamin B-6. Comparing participants with the *MTHFD1L *rs17080689 *CA *genotype to the *CC/AA *genotype, mean LINE-1 element methylation was 0.6 SD higher at low B-6, 0.2 SD higher at median B-6, and 0.4 SD lower at high B-6.

## Discussion

We investigated sequence variation in a network of candidate genes involved in one-carbon metabolism in relation to plasma total homocysteine and two measures of global genomic DNA methylation (Alu, LINE-1).

Genes involved in absorption and transport had the most statistically significant associations with the homocysteine phenotype; about 30-40% of the association was mediated through plasma folate and vitamin B-6 and B-12 levels. For the Alu-element methylation phenotype, the top hits were in genes involved in mitochondrial metabolism, nuclear metabolism, and methylation/homocysteine metabolism. For the LINE-1 methylation phenotype, the top SNP was in a gene in the methylation/homocysteine pathway. There was no evidence that nutrient biomarkers mediated the association of SNPs with the methylation phenotypes.

The set of genes represented in the top hits was unique to each phenotype, although pleiotropy was identified for plasma homocysteine and Alu element methylation involving the *MTHFD1L *and sarcosine dehydrogenase (*SARDH*) genes.

### Plasma total homocysteine phenotype

*SLC19A1*. There were FDR-significant associations between 3 SNPs in the *SLC19A1 *gene and plasma total homocysteine; the direction and magnitude of association were similar. Thus, each copy of the coding nonsynonymous rs1051266 *A *allele, the 5'region rs1131596 *C *allele, and the intronic rs4819130 *C *allele was associated with about a 5.0% increase in plasma homocysteine. HapMap plots indicate high LD across the *SLC19A1 *gene, thus the three SNPs may represent a single effect. The *SLC19A1 *gene encodes a transporter involved in folate and thiamine uptake and may play a role in intracellular folate distribution [21]. Transporter expression may be regulated by folate status [21]. About half of the association of these three *SLC19A1 *SNPs with homocysteine was mediated by plasma folate and vitamins B-6/B-12. The nonsynonymous *SLC19A1 *rs1051266 SNP was previously associated with blood folate levels [22,23], and risk of intracranial aneurysm [24], but not with homocysteine [23,25] or abdominal aortic aneurysm [25]. The 5' region *SLC19A1 *rs1131596 SNP was associated with reduced RBC folate levels in coronary artery disease patients and decreased SLC19A1 protein expression [26,27]. Genetic variation in *SLC19A1 *may influence homocysteine levels, mediated by changes in nutrient biomarkers.

*FTCD*. The intronic *FTCD *rs2277820 SNP was associated with plasma total homocysteine. The *CT *genotype group was 7.2% higher on plasma total homocysteine vs. the *CC/TT *group. *FTCD *encodes a Golgi-associated enzyme involved in the production of 5,10-methenyl-tetrahydrofolate (THF) [1]. Based on HapMap LD patterns the association with the intronic rs2277820 SNP may proxy variation elsewhere in the gene. Mutations in *FTCD *are associated with inherited disorders of folate metabolism [28]. 29% of the association between rs2277820 and homocysteine was mediated through plasma folate and vitamins B-6/B-12.

*SLC19A3*. An FDR-significant association was identified between the intronic rs13007334 SNP in *SLC19A3 *and plasma total homocysteine. The *CT *genotype group was 6.9% higher on plasma total homocysteine vs. the *CC/TT *group. The *SLC19A3 *gene belongs to the folate transporter family and encodes a thiamine transporter [21]. Although SLC19A3 is not known to transport folate or vitamins B-6/B-12, 34% of the SNP--homocysteine association was mediated by these nutrients. No prior reports link *SLC19A3 *to biochemical or disease phenotypes, and a biological basis for the link to thiamine metabolism could not be identified.

The variability in homocysteine explained by the model containing the set of the 3 most significant nonredundant SNP hits was 3.6%, a small proportion of the estimated > 50% heritability in homocysteine [29,30], and similar to the proportion explained by age and smoking together.

There were four FDR-significant interactions between studied SNPs and *MTHFR *rs1801133 (Additional file 4); the most statistically significant was for the *ALDH1L1 *rs2305230 SNP. In participants with the *ALDH1L1 *rs2305230 *AA *genotype, men with 1 copy of the *MTHFR *rs1801133 *T *allele had plasma homocysteine 64% higher than men with no copies. However, among participants with the *ALDH1L1 *rs2305230 *AC/CC *genotype, men with 1 copy of the *MTHFR *rs1801133 *T *allele had plasma homocysteine 2.1% lower than men with no copies.

There were no FDR-significant interactions between studied SNPs and plasma folate, vitamin B-6, or vitamin B-12 for the plasma homocysteine phenotype. The null results may be due to an overly conservative FDR significance threshold, network compensation for genetic and nutritional stresses, or inadequate power to evaluate interactions involving low MAF SNPs; also, the folate status for men in the NAS was relatively high in comparison to national averages as reported in Pfeiffer et al [31], and SNP--nutrient interactions may be attenuated in this range of folate status. The *MTHFR *rs1801133 SNP, which is expected to interact with folate in predicting the homocysteine phenotype, had a nonsignificant interaction in these data (nominal P_interaction _= 0.0578), but the association of *MTHFR *with homocysteine was stronger at lower concentrations of plasma folate (data not shown).

A cluster of SNP--vitamin B-6 interactions was noted for variants in the *CBS *gene, but the P values for these interaction terms were about 0.1 and did not reach thresholds set prior to the analysis. These findings suggest that interactions between vitamin B-6 and genetic variants in the *SHMT1 *and *CBS *genes may only be evident with very low vitamin B-6 status, which is consistent with previous work [32,33]. A systematic review of literature published prior to August, 2009 revealed only one report of a statistically significant interaction between genetic variation in *SHMT1 *(rs1979277) and B-6 [34].

### Global genomic DNA methylation phenotype (Alu elements)

There were no FDR-significant main effect associations for the Alu element methylation outcome. None of the SNP--folate or SNP--vitamin B-12 interaction terms reached FDR significance thresholds. Given that the Alu phenotype was measured after the introduction of mandatory folate fortification in the U.S., findings may be limited. Three FDR-significant SNP--vitamin B-6 interactions were identified, including two intronic SNPs in the *AMT *gene (rs1464567 and rs1464566) and one intronic SNP in the *DNMT3B *gene (rs1883729). The *AMT *gene encodes an enzyme that functions in the vitamin B-6-dependent mitochondrial glycine cleavage system [35]. B-6 interactions involving SNPs in *GLDC *were among the top nominally significant hits for the homocysteine and Alu methylation phenotypes, but did not reach FDR-significance. The *DNMT3B *gene encodes a DNA methyltransferase enzyme that is localized to the nucleus, developmentally regulated, and functions to establish *de novo *methylation patterns [36,37]; *DNMT3B *expression is associated with cancer [36-38]. Although cell culture studies have not supported Alu elements as *DNMT3B *targets [36,39] in both *in vitro *and *in vivo *models, DNMT3b protein levels were down-regulated by B vitamin deficiency (deficiency of folate, B-6, and B-12 together), *de novo *methylation was suppressed both *in vitro *and *in vivo *under conditions of B vitamin deficiency[40], and S-adenosylmethionine levels were markedly decreased in response to lowered B-6 concentrations in culture medium [32] consistent with the direction of association observed here.

### Global genomic DNA methylation phenotype (LINE-1 elements)

There were no FDR-significant associations observed for the LINE-1 methylation phenotype. There were no FDR-significant interactions between SNPs and folate or vitamin B-12; the measurement of LINE-1 in Normative Aging Study men took place after the introduction of mandatory folate fortification in the U.S., and limited variation may have limited findings. A single SNP--vitamin B-6 interaction was significant at the FDR threshold for the intronic rs17080689 in the *MTHFD1L *gene. The *MTHFD1L *gene product functions downstream from the vitamin B-6-dependent glycine cleavage system [41] and intronic variation in *MTHFD1L *was previously associated with CVD [42].

## Conclusions

Strengths of the present study include investigation of a large cohort with homocysteine data collected prior to the introduction of mandatory folate-fortification in the U.S. Also, SNP selection for the genotyping assay reflected functional, LD, and physical coverage of genes. Using a systematic approach, we identified the best genetic models for each SNP, then tested single SNPs, the interaction of each SNP with *MTHFR *rs1801133, and the interaction of each SNP with folate, vitamin B-6 and vitamin B-12. Findings were corrected for multiple comparisons and those surpassing the FDR threshold were discussed in more detail. Weaknesses of the study include the fact that methylation (but not homocysteine) measures were collected after the introduction of mandatory folate fortification in the U.S., which may have limited variation in B-vitamin status. Information on additional nutrients such as choline would have allowed a more complete evaluation of gene-nutrient interactions. S-adenosylhomocysteine and/or the ratio of S-adenosylmethionine to S-adenosylhomocysteine are likely to be more sensitive indicators of vascular disease risk than homocysteine [43,44], but were not measured. The methylation phenotypes studied here are believed to be an adequate proxy of genome-wide DNA methylation. Gene-specific methylation data was not available; furthermore, because the folate-mediated one-carbon network functions to generate cellular methylation potential and thus contributes to numerous methylation reactions, it may be more appropriate to evaluate folate network genetic variation in relation to global measures of methylation. Finally, due to genotyping failure, some key variants could not be analyzed, for example rs6922269 in *MTHFD1L *[42], although proxies were selected purposefully to address this limitation.

The most significant hits for the homocysteine and methylation outcomes reflected genes involved in the generation of one-carbon units, including *SLC19A1 *and *FTCD*. Because a unique set of genes was identified for each phenotype, and because the top hits could not be predicted on the basis of hypothesized impact on cellular methylation potential, this work suggests that not all folate effects are mediated through the ratio of S-adenosylmethionine to S-adenosylhomocysteine. Thus, beyond the well-described *MTHFR *rs1801133 SNP, polymorphisms in other genes make important contributions to homocysteine and global genomic DNA methylation phenotypes. Furthermore, some associations are sensitive to nutritional status of B vitamins. Future work should continue to include a broad evaluation of one-carbon network genetic and nutritional variation in unfortified or pre-fortification populations and extend these findings for CVD biomarkers to an investigation of CVD phenotypes.

## Abbreviations

% 5-meC: percentage of methylated cytosines; 3': 3' region; 5': 5' region; A: Additive; *AHCY*: Adenosylhomocysteinase; *AHCYL1*: Adenosylhomocysteinase-like 1; *AHCYL2*: Adenosylhomocysteinase-like 2, *KIAA0828*; *ALDH1L1*: Aldehyde dehydrogenase 1 family: member L1; *AMT*: Aminomethyltransferase; *ATIC*: 5-aminoimidazole-4-carboxamide ribonucleotide formyltransferase/IMP cyclohydrolase; *BHMT*: Betaine-homocysteine S-methyltransferase; *CBS*: Cystathionine-beta-synthase; *CTH*: Cystathionase (cystathionine gamma-lyase); *CELF1*: *CUGBP*: Elav-like family member 1; CEPH: Centre d'Etude du Polymorphisme Humain; CV: coefficient of variation; CN: Coding nonsynonymous; CS: Coding synonymous; CVD: cardiovascular disease; df: degrees of freedom; *DHFR*: Dihydrofolate reductase; *DMGDH*: Dimethylglycine dehydrogenase; *DNMT1*: DNA (cytosine-5-)-methyltransferase 1; *DNMT3A*: DNA (cytosine-5-)-methyltransferase 3 alpha; *DNMT3B*: DNA (cytosine-5-)-methyltransferase 3 beta; D: Dominant; FDR: False Discovery Rate; *FOLH1*: Folate hydrolase (prostate-specific membrane antigen) 1; *FOLR1*: Folate receptor 1 (adult); *FOLR2*: Folate receptor 2 (fetal); *FOLR3*: Folate receptor 3 (gamma); *FPGS*: Folylpolyglutamate synthase; *FTCD*: Formiminotransferase cyclodeaminase; *FTH1*: Ferritin: heavy polypeptide 1; *GART*: Phosphoribosylglycinamide formyltransferase: phosphoribosylglycinamide synthetase: phosphoribosylaminoimidazole synthetase; *GCSH*: Glycine cleavage system protein H (aminomethyl carrier); *GGH*: Gamma-glutamyl hydrolase (conjugase: folylpolygammaglutamyl hydrolase); *GLDC*: Glycine dehydrogenase (decarboxylating); *GNMT*: Glycine N-methyltransferase; *HSPA8*: Heat shock 70 kDa protein 8; HWE: Hardy-Weinberg equilibrium; I: Intronic; LRT: likelihood ratio test; LD: linkage disequilibrium; MAF: minor allele frequency; *MARS*: Methionyl-tRNA synthetase; *MAT1A*: Methionine adenosyltransferase I: alpha; *MAT2A*: Methionine adenosyltransferase II: alpha; *MAT2B*: Methionine adenosyltransferase II: beta; M.E.: Main effect; *MTHFD1*: Methylenetetrahydrofolate dehydrogenase (NADP+ dependent) 1: methenyltetrahydrofolate cyclohydrolase: formyltetrahydrofolate synthetase; *MTHFD1L*: Methylenetetrahydrofolate dehydrogenase (NADP+ dependent) 1-like; *MTHFD2*: Methylenetetrahydrofolate dehydrogenase (NADP+ dependent) 2: methenyltetrahydrofolate cyclohydrolase; *MTHFR*: Methylenetetrahydrofolate reductase (NADPH); *MTHFS*: 5,10-methenyltetrahydrofolate synthetase (5-formyltetrahydrofolate cyclo-ligase); *MTR*: 5-methyltetrahydrofolate-homocysteine methyltransferase; *MTRR*: 5-methyltetrahydrofolate-homocysteine methyltransferase reductase; NAS: Normative Aging Study; O: Overdominant; PLP: pyridoxal-5'-phosphate; R: Recessive; *SARDH*: Sarcosine dehydrogenase; *SHMT1*: Serine hydroxymethyltransferase 1 (soluble); *SHMT2*: Serine hydroxymethyltransferase 2 (mitochondrial); *SLC19A1*: Solute carrier family 19 (folate transporter): member 1; *SLC19A2*: Solute carrier family 19 (thiamine transporter): member 2; *SLC19A3*: Solute carrier family 19: member 3; *SLC25A32*: Solute carrier family 25: member 32; *SLC46A1*: Solute carrier family 46 (folate transporter): member 1; SNP: single nucleotide polymorphism; *TCN1*: Transcobalamin I (vitamin B-12 binding protein: R binder family); *TCN2*: Transcobalamin II; THF: tetrahydrofolate; *TYMS*: Thymidylate synthetase; *UBE2I*: Ubiquitin-conjugating enzyme E2I (UBC9 homolog: yeast); *UBE2N*: Ubiquitin-conjugating enzyme E2N (UBC13 homolog: yeast); VA: Veterans' Administration.

## Competing interests

The authors declare that they have no competing interests.

## Authors' contributions

SMW, PAC, AAL, STW, JMG, KLT, AB, and JS designed research; SMW, PJS, AGC, MTW, VB and PAC conducted research; SMW and PAC analyzed data; SMW, PAC, AGC, PJS, and MTW wrote the paper, and PAC had primary responsibility for all work and final content. All authors read and approved the final manuscript.

## Pre-publication history

The pre-publication history for this paper can be accessed here:

http://www.biomedcentral.com/1471-2350/12/150/prepub

## Supplementary Material

Additional file 1 **52 genes in the folate-mediated one-carbon pathway**. Click here for file

Additional file 2 **330 folate-related SNPs assayed in men in the Normative Aging Study**. Click here for file

Additional file 3 **Supplemental methods**. Click here for file

Additional file 4 **Epistatic interactions with the *MTHFR *rs1801133 SNP and plasma homocysteine**. The most statistically significant associations (FDR-adjusted Likelihood Ratio Test P ≤ 0.2) for SNP by *MTHFR *rs1801133 interactions in relation to the plasma homocysteine phenotype for men in the Normative Aging Study. Click here for file

Additional file 5 **Gene-nutrient interactions and plasma homocysteine**. The most statistically significant associations (P ≤ 0.02) for SNP by nutrient interactions in relation to the plasma homocysteine phenotype for men in the Normative Aging Study. Click here for file

Additional file 6 **Gene-nutrient interactions and Alu element methylation**. The most statistically significant associations (P ≤ 0.02) for SNP by nutrient interactions in relation to the global genomic DNA methylation phenotype (Alu elements) for men in the Normative Aging Study. Click here for file

Additional file 7 **Gene-nutrient interactions and LINE-1 element methylation**. The most statistically significant associations (P ≤ 0.02) for SNP by nutrient interactions in relation to the global genomic DNA methylation phenotype (LINE-1 elements) for men in the Normative Aging Study Click here for file
