# Supplementary material for: Folate network genetic variation, plasma homocysteine, and global genomic methylation content: a genetic association study
Source: BMC Med Genet. 2011 Nov 21;12:150. doi: 10.1186/1471-2350-12-150 (PMC3266217; doi:10.1186/1471-2350-12-150)
Supplement: Additional file 1 — 52 genes in the folate-mediated one-carbon pathway. [file 1471-2350-12-150-S1.DOC]

# Folate network genetic variation, plasma homocysteine, and global genomic methylation content: a genetic association study

Susan M Wernimont^1^, Andrew G Clark^2^, Patrick J Stover^1^, Martin T Wells^3^, Augusto A Litonjua^4^, Scott T Weiss^4^, J Michael Gaziano^5^, Katherine L Tucker^6^, Andrea Baccarelli^7,8^, Joel Schwartz^7^, Valentina Bollati^8^, and Patricia A Cassano^9§^

^1^Division of Nutritional Sciences, Cornell University, Ithaca, NY, USA

^2^Department of Molecular Biology & Genetics, Cornell University, Ithaca, NY, USA

^3^Department of Biological Statistics & Computational Biology, Cornell, Ithaca, NY, USA

^4^Channing Laboratory, Brigham and Women’s Hospital, and Harvard Medical School, Boston, MA, USA

^5^Division of Aging, Brigham & Women's Hospital, Boston, MA, USA

^6^Department of Health Sciences, Northeastern University, Boston, MA, USA

^7^Departments of Environmental Health and Epidemiology, Harvard University, Boston, MA, USA

^8^Center of Molecular and Genetic Epidemiology, Department of Environmental and Occupational Health, Università degli Studi di Milano and IRCCS Fondazione Ca’ Granda Ospedale Maggiore Policlinico, Milan, Italy

^9^209 Savage Hall, Division of Nutritional Sciences, Cornell University, Ithaca, NY, USA

^§^Corresponding author

Email addresses:

SMW: [smw38@cornell.edu](mailto:smw38@cornell.edu)

AGC: [ac347@cornell.edu](mailto:ac347@cornell.edu)

PJS: [pjs13@cornell.edu](mailto:pjs13@cornell.edu)

MTW: [mtw1@cornell.edu](mailto:mtw1@cornell.edu)

AAL: [ALITONJUA@PARTNERS.ORG](mailto:ALITONJUA@PARTNERS.ORG)

STW: [scott.weiss@channing.harvard.edu](mailto:scott.weiss@channing.harvard.edu)

JMG: [jmgaziano@partners.org](mailto:jmgaziano@partners.org)

KLT: [KL.Tucker@neu.edu](mailto:KL.Tucker@neu.edu)

AB: [abaccare@hsph.harvard.edu](mailto:abaccare@hsph.harvard.edu)

JS: [JSCHWRTZ@hsph.harvard.edu](mailto:JSCHWRTZ@hsph.harvard.edu)

VB: [abaccare@hsph.harvard.edu](mailto:abaccare@hsph.harvard.edu)

PAC: [pac6@cornell.edu](mailto:pac6@cornell.edu)

**Additional file 1** 52 genes in the folate-mediated one-carbon pathway

| **Gene Symbol** | **Gene Name** | **Gene ID** |
| --- | --- | --- |
| *AHCY* | Adenosylhomocysteinase | 191 |
| *AHCYL1* | Adenosylhomocysteinase-like 1 | 10768 |
| *AHCYL2* | Adenosylhomocysteinase-like 2, KIAA0828 | 23382 |
| *ALDH1L1* | Aldehyde dehydrogenase 1 family, member L1 | 10840 |
| *AMT* | Aminomethyltransferase | 275 |
| *ATIC* | 5-aminoimidazole-4-carboxamide ribonucleotide formyltransferase/IMP cyclohydrolase | 471 |
| *BHMT* | Betaine-homocysteine S-methyltransferase | 635 |
| *CBS* | Cystathionine-beta-synthase | 875 |
| *CTH* | Cystathionase (cystathionine gamma-lyase) | 1491 |
| *CELF1* | CUGBP, Elav-like family member 1 | 10658 |
| *DHFR* | Dihydrofolate reductase | 1719 |
| *DMGDH* | Dimethylglycine dehydrogenase | 29958 |
| *DNMT1* | DNA (cytosine-5-)-methyltransferase 1 | 1786 |
| *DNMT3A* | DNA (cytosine-5-)-methyltransferase 3 alpha | 1788 |
| *DNMT3B* | DNA (cytosine-5-)-methyltransferase 3 beta | 1789 |
| *FOLH1* | Folate hydrolase (prostate-specific membrane antigen) 1 | 2346 |
| *FOLR1* | Folate receptor 1 (adult) | 2348 |
| *FOLR2* | Folate receptor 2 (fetal) | 2350 |
| *FOLR3* | Folate receptor 3 (gamma) | 2352 |
| *FPGS* | Folylpolyglutamate synthase | 2356 |
| *FTCD* | Formiminotransferase cyclodeaminase | 10841 |
| *FTH1* | Ferritin, heavy polypeptide 1 | 2495 |
| *GART* | Phosphoribosylglycinamide formyltransferase, phosphoribosylglycinamide synthetase, phosphoribosylaminoimidazole synthetase | 2618 |
| *GCSH* | Glycine cleavage system protein H (aminomethyl carrier) | 2653 |
| *GGH* | Gamma-glutamyl hydrolase (conjugase, folylpolygammaglutamyl hydrolase) | 8836 |
| *GLDC* | Glycine dehydrogenase (decarboxylating) | 2731 |
| *GNMT* | Glycine N-methyltransferase | 27232 |
| *HSPA8* | Heat shock 70kDa protein 8 | 3312 |
| *MARS* | Methionyl-tRNA synthetase | 4141 |
| *MAT1A* | Methionine adenosyltransferase I, alpha | 4143 |
| *MAT2A* | Methionine adenosyltransferase II, alpha | 4144 |
| *MAT2B* | Methionine adenosyltransferase II, beta | 27430 |
| *MTHFD1* | Methylenetetrahydrofolate dehydrogenase (NADP+ dependent) 1, methenyltetrahydrofolate cyclohydrolase, formyltetrahydrofolate synthetase | 4522 |
| *MTHFD1L* | Methylenetetrahydrofolate dehydrogenase (NADP+ dependent) 1-like | 25902 |
| *MTHFD2* | Methylenetetrahydrofolate dehydrogenase (NADP+ dependent) 2, methenyltetrahydrofolate cyclohydrolase | 10797 |
| *MTHFR* | Methylenetetrahydrofolate reductase (NAD(P)H) | 4524 |
| *MTHFS* | 5,10-methenyltetrahydrofolate synthetase (5-formyltetrahydrofolate cyclo-ligase) | 10588 |
| *MTR* | 5-methyltetrahydrofolate-homocysteine methyltransferase | 4548 |
| *MTRR* | 5-methyltetrahydrofolate-homocysteine methyltransferase reductase | 4552 |

**Additional file 1** 52 genes in the folate-mediated one-carbon pathway (cont’d)

| **Gene Symbol** | **Gene Name** | **Gene ID** |
| --- | --- | --- |
| *SARDH* | Sarcosine dehydrogenase | 1757 |
| *SHMT1* | Serine hydroxymethyltransferase 1 (soluble) | 6470 |
| *SHMT2* | Serine hydroxymethyltransferase 2 (mitochondrial) | 6472 |
| *SLC19A1* | Solute carrier family 19 (folate transporter), member 1 | 6573 |
| *SLC19A2* | Solute carrier family 19 (thiamine transporter), member 2 | 10560 |
| *SLC19A3* | Solute carrier family 19, member 3 | 80704 |
| *SLC25A32* | Solute carrier family 25, member 32 | 81034 |
| *SLC46A1* | Solute carrier family 46 (folate transporter), member 1 | 113235 |
| *TCN1* | Transcobalamin I (vitamin B-12 binding protein, R binder family) | 6947 |
| *TCN2* | Transcobalamin II | 6948 |
| *TYMS* | Thymidylate synthetase | 7298 |
| *UBE2I* | Ubiquitin-conjugating enzyme E2I (UBC9 homolog, yeast) | 7329 |
| *UBE2N* | Ubiquitin-conjugating enzyme E2N (UBC13 homolog, yeast) | 7334 |
