# Supplementary material for: Folate network genetic variation, plasma homocysteine, and global genomic methylation content: a genetic association study
Source: BMC Med Genet. 2011 Nov 21;12:150. doi: 10.1186/1471-2350-12-150 (PMC3266217; doi:10.1186/1471-2350-12-150)
Supplement: Additional file 2 — 330 folate-related SNPs assayed in men in the Normative Aging Study. [file 1471-2350-12-150-S2.DOC]

# Folate network genetic variation, plasma homocysteine, and global genomic methylation content: a genetic association study

Susan M Wernimont^1^, Andrew G Clark^2^, Patrick J Stover^1^, Martin T Wells^3^, Augusto A Litonjua^4^, Scott T Weiss^4^, J Michael Gaziano^5^, Katherine L Tucker^6^, Andrea Baccarelli^7,8^, Joel Schwartz^7^, Valentina Bollati^8^, and Patricia A Cassano^9§^

^1^Division of Nutritional Sciences, Cornell University, Ithaca, NY, USA

^2^Department of Molecular Biology & Genetics, Cornell University, Ithaca, NY, USA

^3^Department of Biological Statistics & Computational Biology, Cornell, Ithaca, NY, USA

^4^Channing Laboratory, Brigham and Women’s Hospital, and Harvard Medical School, Boston, MA, USA

^5^Division of Aging, Brigham & Women's Hospital, Boston, MA, USA

^6^Department of Health Sciences, Northeastern University, Boston, MA, USA

^7^Departments of Environmental Health and Epidemiology, Harvard University, Boston, MA, USA

^8^Center of Molecular and Genetic Epidemiology, Department of Environmental and Occupational Health, Università degli Studi di Milano and IRCCS Fondazione Ca’ Granda Ospedale Maggiore Policlinico, Milan, Italy

^9^209 Savage Hall, Division of Nutritional Sciences, Cornell University, Ithaca, NY, USA

^§^Corresponding author

Email addresses:

SMW: [smw38@cornell.edu](mailto:smw38@cornell.edu)

AGC: [ac347@cornell.edu](mailto:ac347@cornell.edu)

PJS: [pjs13@cornell.edu](mailto:pjs13@cornell.edu)

MTW: [mtw1@cornell.edu](mailto:mtw1@cornell.edu)

AAL: [ALITONJUA@PARTNERS.ORG](mailto:ALITONJUA@PARTNERS.ORG)

STW: [scott.weiss@channing.harvard.edu](mailto:scott.weiss@channing.harvard.edu)

JMG: [jmgaziano@partners.org](mailto:jmgaziano@partners.org)

KLT: [KL.Tucker@neu.edu](mailto:KL.Tucker@neu.edu)

AB: [abaccare@hsph.harvard.edu](mailto:abaccare@hsph.harvard.edu)

JS: [JSCHWRTZ@hsph.harvard.edu](mailto:JSCHWRTZ@hsph.harvard.edu)

VB: [abaccare@hsph.harvard.edu](mailto:abaccare@hsph.harvard.edu)

PAC: [pac6@cornell.edu](mailto:pac6@cornell.edu)

**Additional file 2** 330 folate-related SNPs assayed in men in the Normative Aging

Study.

| **Gene** | **SNP Name** | **Gene** | **SNP Name** | **Gene** | **SNP Name** |
| --- | --- | --- | --- | --- | --- |
| *AHCY* | AHCYC34T | *BHMT* | rs558133 | *DHFR* | rs380691 |
| *AHCY* | rs1205366 | *BHMT* | rs567754 | *DHFR* | rs836822 |
| *AHCY* | rs13043752 | *BHMT* | rs585800 | *DMGDH* | rs1805073 |
| *AHCY* | rs819133 | *BHMT* | rs7700970 | *DMGDH* | rs1805074 |
| *AHCY* | rs819146 | *CBS* | rs11203172 | *DMGDH* | rs2253262 |
| *AHCY* | rs819148 | *CBS* | rs11701048 | *DMGDH* | rs28326 |
| *AHCY* | rs819155 | *CBS* | rs12329764 | *DMGDH* | rs532964 |
| *AHCY* | rs819159 | *CBS* | rs1788484 | *DMGDH* | rs644191 |
| *AHCYL1* | rs186724 | *CBS* | rs1789953 | *DNMT1* | rs2228612^a^ |
| *AHCYL1* | rs2298116 | *CBS* | rs1801181 | *DNMT1* | rs11880388 |
| *AHCYL1* | rs333079 | *CBS* | rs2014564 | *DNMT1* | rs2162560 |
| *AHCYL1* | rs3768480 | *CBS* | rs2124458 | *DNMT1* | rs2228611 |
| *AHCYL1* | rs720917 | *CBS* | rs234704 | *DNMT1* | rs8101626 |
| *AHCYL2* | rs1665105 | *CBS* | rs234705 | *DNMT3A* | rs11678631 |
| *AHCYL2* | rs4731569 | *CBS* | rs234706 | *DNMT3A* | rs11695471 |
| *AHCYL2* | rs6467233 | *CBS* | rs234709 | *DNMT3A* | rs1550117 |
| *AHCYL2* | rs6467244 | *CBS* | rs234711 | *DNMT3A* | rs6546045 |
| *AHCYL2* | rs7788327 | *CBS* | rs6586281 | *DNMT3A* | rs6733868 |
| *ALDH1L1* | rs11715574 | *CBS* | rs6586282 | *DNMT3A* | rs7578575 |
| *ALDH1L1* | rs1823213 | *CBS* | rs706209 | *DNMT3B* | rs1883729 |
| *ALDH1L1* | rs1868138 | *CBS* | rs760124 | *DNMT3B* | rs2424914 |
| *ALDH1L1* | rs2305230 | *CTH* | rs1021737 | *DNMT3B* | rs2424922 |
| *ALDH1L1* | rs3772414 | *CTH* | rs535112 | *DNMT3B* | rs6058869 |
| *ALDH1L1* | rs3772424 | *CTH* | rs648743 | *DNMT3B* | rs6058891 |
| *ALDH1L1* | rs4646745 | *CTH* | rs663465 | *DNMT3B* | rs6058896 |
| *ALDH1L1* | rs4646750 | *CTH* | rs663649 | *FOLH1* | rs16906158 |
| *ALDH1L1* | rs4646760 | *CTH* | rs681475 | *FOLH1* | rs202673 |
| *AMT* | rs11922013 | *CUGBP1* | rs2242081 | *FOLH1* | rs202676 |
| *AMT* | rs1464566 | *CUGBP1* | rs4752843 | *FOLH1* | rs664584 |
| *AMT* | rs1464567 | *CUGBP1* | rs7102372 | *FOLR1* | rs2071010 |
| *AMT* | rs8897 | *CUGBP1* | rs7933019 | *FOLR1* | rs9282688 |
| *ATIC* | rs1997059 | *DHFR* | rs12517451 | *FOLR2* | rs2298444 |
| *ATIC* | rs2372536 | *DHFR* | rs1382540 | *FOLR2* | rs514933 |
| *ATIC* | rs3821353 | *DHFR* | rs1643650 | *FOLR3* | rs11235449 |
| *ATIC* | rs4672768 | *DHFR* | rs1643659 | *FOLR3* | rs7926875 |
| *ATIC* | rs7585489 | *DHFR* | rs1650697 | *FPGS* | rs10106 |
| *BHMT* | rs10037045 | *DHFR* | rs1650723 | *FPGS* | rs41319447 |
| *BHMT* | rs16876512 | *DHFR* | rs1677666 | *FPGS* | rs4451422 |
| *BHMT* | rs506500 | *DHFR* | rs2560424 | *FTCD* | rs16978930 |

**Additional file 2** 330 folate-related SNPs (cont’d)

| **Gene** | **SNP Name** | **Gene** | **SNP Name** | **Gene** | **SNP Name** |
| --- | --- | --- | --- | --- | --- |
| *FTCD* | rs2277820 | *HSPA8* | rs4936770 | *MTHFD1L* | rs4869954 |
| *FTH1* | rs17156609 | *MARS* | rs1678537 | *MTHFD1L* | rs4869955 |
| *FTH1* | rs17185413 | *MARS* | rs496245 | *MTHFD1L* | rs509474 |
| *FTH1* | rs1800009 | *MARS* | rs899653 | *MTHFD1L* | rs524732 |
| *FTH1* | rs1801621 | *MAT1A* | rs10788546 | *MTHFD1L* | rs538017 |
| *FTH1* | rs2073588 | *MAT1A* | rs1143694 | *MTHFD1L* | rs6902664 |
| *GART* | rs1804385 | *MAT1A* | rs17677908 | *MTHFD1L* | rs6910267 |
| *GART* | rs2027592 | *MAT1A* | rs1819684 | *MTHFD1L* | rs742832 |
| *GART* | rs4817580 | *MAT1A* | rs1985908 | *MTHFD1L* | rs7746991 |
| *GART* | rs8788^b^ | *MAT1A* | rs2993763 | *MTHFD1L* | rs7765521 |
| *GART* | rs8971 | *MAT2A* | rs1078004 | *MTHFD1L* | rs7770982 |
| *GCSH* | rs11866124 | *MAT2A* | rs1446667 | *MTHFD1L* | rs803422 |
| *GCSH* | rs1563072 | *MAT2A* | rs2028900 | *MTHFD1L* | rs803446 |
| *GCSH* | rs4889233 | *MAT2A* | rs2043675 | *MTHFD1L* | rs803447 |
| *GCSH* | rs8177876 | *MAT2B* | rs10515861 | *MTHFD1L* | rs803454 |
| *GCSH* | rs8177940 | *MAT2B* | rs17061795 | *MTHFD1L* | rs803455 |
| *GGH* | rs11545078 | *MAT2B* | rs4869087 | *MTHFD1L* | rs803456 |
| *GGH* | rs11995525 | *MAT2B* | rs6882306 | *MTHFD1L* | rs803466 |
| *GGH* | rs12544045 | *MAT2B* | rs7733775 | *MTHFD1L* | rs9478162 |
| *GGH* | rs3780126 | *MTHFD1* | rs1076991 | *MTHFD1L* | rs9478908 |
| *GGH* | rs4617146 | *MTHFD1* | rs11627525 | *MTHFD1L* | rs997429 |
| *GLDC* | rs10975681 | *MTHFD1* | rs17751556 | *MTHFD2* | rs1667627 |
| *GLDC* | rs1755617 | *MTHFD1* | rs1950902 | *MTHFD2* | rs7340453 |
| *GLDC* | rs1821892 | *MTHFD1* | rs2281603 | *MTHFR* | rs12121543 |
| *GLDC* | rs1929933 | *MTHFD1* | rs3783728 | *MTHFR* | rs13306556 |
| *GLDC* | rs2118653 | *MTHFD1* | rs8003379 | *MTHFR* | rs1537516 |
| *GLDC* | rs3902970 | *MTHFD1* | rs8003567 | *MTHFR* | rs17367629 |
| *GLDC* | rs4237166 | *MTHFD1* | rs8012229 | *MTHFR* | rs17421462 |
| *GLDC* | rs4629927 | *MTHFD1L* | rs1047665 | *MTHFR* | rs1801133 |
| *GLDC* | rs7049056 | *MTHFD1L* | rs1076746 | *MTHFR* | rs1994798 |
| *GLDC* | rs7848919 | *MTHFD1L* | rs11754661 | *MTHFR* | rs3737965 |
| *GNMT* | rs1051218 | *MTHFD1L* | rs12201472 | *MTHFR* | rs4846049 |
| *GNMT* | rs11752813 | *MTHFD1L* | rs1474787 | *MTHFR* | rs6541003 |
| *GNMT* | rs2296804 | *MTHFD1L* | rs17080689 | *MTHFR* | rs1801131 |
| *GNMT* | rs2296805 | *MTHFD1L* | rs17349743 | *MTHFS* | rs2586167 |
| *GNMT* | rs736158 | *MTHFD1L* | rs17354394 | *MTHFS* | rs2733106 |
| *HSPA8* | rs11218941 | *MTHFD1L* | rs1738574 | *MTHFS* | rs6495450 |
| *HSPA8* | rs1136141 | *MTHFD1L* | rs2295084 | *MTHFS* | rs7177659 |
| *HSPA8* | rs1461496 | *MTHFD1L* | rs4869953 | *MTHFS* | rs8923 |

**Additional file 2** 330 folate-related SNPs (cont’d)

| **Gene** | **SNP Name** | **Gene** | **SNP Name** | **Gene** | **SNP Name** |
| --- | --- | --- | --- | --- | --- |
| *MTR* | rs2229276^c^ | *SHMT1* | rs2461838 | *TCN1* | rs526934 |
| *MTR* | rs1050996 | *SHMT1* | rs4924750 | *TCN1* | rs557564 |
| *MTR* | rs10925260 | *SHMT1* | rs643333 | *TCN2* | rs10418 |
| *MTR* | rs1131449^d^ | *SHMT2* | rs28365862 | *TCN2* | rs2301957 |
| *MTR* | rs16834388 | *SHMT2* | rs7301155 | *TCN2* | rs2301958 |
| *MTR* | rs1805087 | *SLC19A1* | rs1051266 | *TCN2* | rs4820021 |
| *MTR* | rs2275566 | *SLC19A1* | rs1051298 | *TCN2* | rs4820886 |
| *MTR* | rs3754255 | *SLC19A1* | rs1131596^e^ | *TCN2* | rs4820887 |
| *MTRR* | rs10380 | *SLC19A1* | rs12482346 | *TCN2* | rs4820889 |
| *MTRR* | rs1532268 | *SLC19A1* | rs2297291 | *TCN2* | rs5749131 |
| *MTRR* | rs161869 | *SLC19A1* | rs4819130 | *TCN2* | rs5749132 |
| *MTRR* | rs162036 | *SLC19A2* | rs17518769 | *TCN2* | rs5753231 |
| *MTRR* | rs1801394 | *SLC19A2* | rs1983546 | *TCN2* | rs740233 |
| *MTRR* | rs1802059 | *SLC19A2* | rs2038024 | *TCN2* | rs757874 |
| *MTRR* | rs2303081 | *SLC19A2* | rs6656822 | *TCN2* | rs9606756 |
| *MTRR* | rs2966952 | *SLC19A3* | rs11694828 | *TCN2* | rs9621049 |
| *MTRR* | rs3776465 | *SLC19A3* | rs13007334 | *TYMS* | rs16948305 |
| *MTRR* | rs7730643 | *SLC19A3* | rs13025803 | *TYMS* | rs2612095 |
| *MTRR* | rs8659 | *SLC19A3* | rs17438244 | *TYMS* | rs2790 |
| *SARDH* | rs129886 | *SLC25A32* | rs1061196 | *TYMS* | rs2853533 |
| *SARDH* | rs129891 | *SLC25A32* | rs17803441 | *TYMS* | rs2853543 |
| *SARDH* | rs129932 | *SLC25A32* | rs3098243 | *TYMS* | rs502396 |
| *SARDH* | rs2073815 | *SLC25A32* | rs3098260 | *TYMS* | rs699517 |
| *SARDH* | rs2073817 | *SLC25A32* | rs3134297 | *UBE2I* | rs11248868 |
| *SARDH* | rs2502741 | *SLC46A1* | rs17719944 | *UBE2I* | rs8052688 |
| *SARDH* | rs4979632 | *SLC46A1* | rs2239907 | *UBE2I* | rs909915 |
| *SARDH* | rs756682 | *SLC46A1* | rs2239908 | *UBE2I* | rs9926094 |
| *SHMT1* | rs12952556 | *TCN1* | rs17154234 | *UBE2N* | rs1483003 |
| *SHMT1* | rs17806489 | *TCN1* | rs2000613 | *UBE2N* | rs4020454 |
| *SHMT1* | rs1979276 | *TCN1* | rs34324219 | *UBE2N* | rs7300607 |
| *SHMT1* | rs1979277 | *TCN1* | rs34528912 | *UBE2N* | rs7309933 |
| *SHMT1* | rs2273028 | *TCN1* | rs519221 | *UBE2N* | rs7311222 |

^a^Formerly known as rs8111085; ^b^Formerly known as rs9984077; ^c^Formerly known as rs16834521;

^d^Formerly known as rs10737812; ^e^Formerly known as rs3177999.
