# Supplementary material for: Folate network genetic variation, plasma homocysteine, and global genomic methylation content: a genetic association study
Source: BMC Med Genet. 2011 Nov 21;12:150. doi: 10.1186/1471-2350-12-150 (PMC3266217; doi:10.1186/1471-2350-12-150)
Supplement: Additional file 3 — Supplemental methods. [file 1471-2350-12-150-S3.DOC]

# Folate network genetic variation, plasma homocysteine, and global genomic methylation content: a genetic association study

Susan M Wernimont^1^, Andrew G Clark^2^, Patrick J Stover^1^, Martin T Wells^3^, Augusto A Litonjua^4^, Scott T Weiss^4^, J Michael Gaziano^5^, Katherine L Tucker^6^, Andrea Baccarelli^7,8^, Joel Schwartz^7^, Valentina Bollati^8^, and Patricia A Cassano^9§^

^1^Division of Nutritional Sciences, Cornell University, Ithaca, NY, USA

^2^Department of Molecular Biology & Genetics, Cornell University, Ithaca, NY, USA

^3^Department of Biological Statistics & Computational Biology, Cornell, Ithaca, NY, USA

^4^Channing Laboratory, Brigham and Women’s Hospital, and Harvard Medical School, Boston, MA, USA

^5^Division of Aging, Brigham & Women's Hospital, Boston, MA, USA

^6^Department of Health Sciences, Northeastern University, Boston, MA, USA

^7^Departments of Environmental Health and Epidemiology, Harvard University, Boston, MA, USA

^8^Center of Molecular and Genetic Epidemiology, Department of Environmental and Occupational Health, Università degli Studi di Milano and IRCCS Fondazione Ca’ Granda Ospedale Maggiore Policlinico, Milan, Italy

^9^209 Savage Hall, Division of Nutritional Sciences, Cornell University, Ithaca, NY, USA

^§^Corresponding author

Email addresses:

SMW: [smw38@cornell.edu](mailto:smw38@cornell.edu)

AGC: [ac347@cornell.edu](mailto:ac347@cornell.edu)

PJS: [pjs13@cornell.edu](mailto:pjs13@cornell.edu)

MTW: [mtw1@cornell.edu](mailto:mtw1@cornell.edu)

AAL: [ALITONJUA@PARTNERS.ORG](mailto:ALITONJUA@PARTNERS.ORG)

STW: [scott.weiss@channing.harvard.edu](mailto:scott.weiss@channing.harvard.edu)

JMG: [jmgaziano@partners.org](mailto:jmgaziano@partners.org)

KLT: [KL.Tucker@neu.edu](mailto:KL.Tucker@neu.edu)

AB: [abaccare@hsph.harvard.edu](mailto:abaccare@hsph.harvard.edu)

JS: [JSCHWRTZ@hsph.harvard.edu](mailto:JSCHWRTZ@hsph.harvard.edu)

VB: [abaccare@hsph.harvard.edu](mailto:abaccare@hsph.harvard.edu)

PAC: [pac6@cornell.edu](mailto:pac6@cornell.edu)

**Additional file 3**

**Supplemental methods**

Study Population

The rate of continued participation of NAS men over the follow-up period from 1961 through 1998 was excellent, with <1% attrition for all causes. As of June 1998, just prior to the homocysteine phenotype measurements, 543 participants (24%) were deceased and ~1600 men (70%) were actively participating in follow-up visits (mean age of 70 y). Participants in the NAS were primarily non-Hispanic males (>98% of the total NAS population) and the small number of men in other racial/ethnic groups precluded separate analyses.

DNA Extraction

Whole-genome amplified samples were quality checked using Taqman genotyping [1] and poor-performing samples were excluded. Of 1,304 participant samples submitted for genotyping, 54.4% were genomic, 45.5% were whole-genome amplified.

SNP Selection

Four databases were used for SNP selection: the National Center for Biotechnology Information (NCBI) dbSNP and PubMed databases, (<http://www.ncbi.nlm.nih.gov/>), the Applied Biosystems Incorporated (ABI) SNPBrowser website ([http://www.allsnps.com](http://www.allsnps.com/)), and the Illumina Assay Design Tool ([www.illumina.com](http://www.illumina.com/) ). SNPs identified from literature searches and functional variants (non-synonymous coding region SNPs and promoter/regulatory region SNPs) were selected preferentially. Next, gene coverage considerations assessed linkage disequilibrium (LD) across the gene and physical coverage of the gene. Adjacent SNPs were selected such that the decay of maximum linkage disequilibrium between the 2 was no more than 33% (≤0.9 LDU between adjacent SNPs, where 1 LDU represents the decay of LD between two SNPs by about 37% of its maximum value when fitted to the Malecot model [2]) whenever possible to ensure sufficient SNP density to adequately represent the LD characteristics of the gene. GoldenGate SNP validation status was considered at each step, and SNPs with a minor allele frequency (MAF) ≥5% in European-ancestry populations were selected where possible although exceptions were made for SNPs with prior evidence of putative function or when no SNPs with MAF ≥5% were available.

SNP Genotyping

Genotype frequencies in controls were compared with those expected in Hardy-Weinberg equilibrium (HWE) and tested with Monte Carlo permutation estimates of exact P-values for HWE using 10,000 permutations. Of 384 SNPs submitted for genotyping, reasons for exclusion included: assay failure (46), monomorphic genotype data (4), minor allele frequencies less than 1% (3), and genotype frequencies out of HWE (1). Both blind duplicates and HapMap Centre d'Etude du Polymorphisme Humain (CEPH) control samples with known genotypes were included in the genotyping assay, and reproducibility rates were excellent (99.99% for blind duplicates, 99.83% for HapMap CEPH controls).

Covariates

Since the time of enrollment participants have had clinical examinations at 3- to 5-year intervals, with a response rate > 90% for mailed questionnaires. Fasting plasma samples were drawn at the VA field site and stored at -80 °C. Plasma samples were transferred to the Jean Mayer USDA Human Nutrition Research Center on Aging, where they were analyzed; the time between blood draw and analysis averaged 1.7 ± 1.2 y as previously described [3]. Plasma nutrient biomarkers were assayed in an unselected subset of stored blood samples as previously described and coefficients of variation (CV) were uniformly excellent, as follows: 4.3% for folate, 5.0% for vitamin B-6, and 4.7% for vitamin B-12 [3].

Phenotype Assessment

Methods for the determination of plasma total homocysteine were published, and the CV for the assay was 4.0% [3]. Alu and LINE-1 transposons were assayed in bisulfite-treated blood leukocyte genomic DNA using highly quantitative polymerase chain reaction –pyrosequencing technology as previously described. The degree of methylation was expressed for both Alu and LINE-1 as the percentage of methylated cytosines over the sum of methylated and unmethylated cytosines (% 5-meC). Each marker was tested in three replicates, and the average was used in all analyses.

Statistical Analyses

Additive, dominant, recessive, and overdominant genetic models of inheritance were considered for each SNP, and the model with the lowest P value in the unadjusted single SNP analysis was chosen as the best model going forward to obtain the best estimate of the genotype—phenotype association. While this affects the P value distribution going forward, no additional multiple testing corrections were applied beyond the FDR given that the P values from the various models are not independent (for example, between additive and dominant models) and because the work is hypothesis-oriented and seeking to nominate candidate genes for further investigation.  FDR adjustment was implemented in SAS using PROC MULTTEST with the FDR option, and can be considered a conditional FDR given the prior step identifying the best genetic model. The *MTHFR* rs1801133 SNP was coded using a model-free dummy variable approach. Where specific genetic models of inheritance led to sparse data for main effects analyses, additive coding was used as the default. A small number of SNPs could not be evaluated in an interaction with *MTHFR* rs1801133, due to sparse data.

To investigate the joint role of nutrition and genetic variation, analyses of the SNP—phenotype association considered the possibility that nutrient biomarkers may affect this association by: 1) contributing to variation (“noise”) in the phenotype due to causal pathways that do not involve the SNP under consideration, 2) mediating the association of the SNP with the phenotype, and 3) modifying the SNP—phenotype association. Because the set of genes under consideration encode enzymes that function as an interconnected network, it was important to account for variation in the phenotype due to causes other than the SNP under consideration; for example, homocysteine is well-known to respond to folate levels, which may be influenced by a variety of factors including diet and genetic variants.

To understand the extent to which nutrient biomarkers mediated the SNP-phenotype association, models adjusted for the nutrient were compared to unadjusted models. Additionally, nutrients may modify the SNP—phenotype association (for example, the association of *MTHFR* *677 C→T* rs1801133 with homocysteine is modified by blood folate levels [4]).

Nutrient residuals (variation in plasma nutrient not associated with SNP) were calculated for each SNP in order to adjust models for variation in the nutrient biomarker that was not directly associated with the SNP of interest. To facilitate description of the genotype—nutrient interaction, a standardized approach was used, as follows: significant SNP—nutrient interactions were evaluated at 3 levels of the centered, log transformed nutrients: the 10^th^ percentile (“low nutrient levels”), the 50^th^ percentile (“median nutrient levels”), and the 90^th^ percentile (“high nutrient levels”).

Reference List

1. Yan J, Feng J, Hosono S, Sommer SS: **Assessment of multiple displacement amplification in molecular epidemiology.** *Biotechniques* 2004, **37:** 136-3.

2. Maniatis N, Collins A, Xu CF, McCarthy LC, Hewett DR, Tapper W *et al*.: **The first linkage disequilibrium (LD) maps: delineation of hot and cold blocks by diplotype analysis.** *Proc Natl Acad Sci U S A* 2002, **99:** 2228-2233.

3. Tucker KL, Qiao N, Scott T, Rosenberg I, Spiro A, III: **High homocysteine and low B vitamins predict cognitive decline in aging men: the Veterans Affairs Normative Aging Study.** *Am J Clin Nutr* 2005, **82:** 627-635.

4. Jacques PF, Bostom AG, Williams RR, Ellison RC, Eckfeldt JH, Rosenberg IH *et al*.: **Relation between folate status, a common mutation in methylenetetrahydrofolate reductase, and plasma homocysteine concentrations.** *Circulation* 1996, **93:** 7-9.
