# Supplementary material for: Folate network genetic variation, plasma homocysteine, and global genomic methylation content: a genetic association study
Source: BMC Med Genet. 2011 Nov 21;12:150. doi: 10.1186/1471-2350-12-150 (PMC3266217; doi:10.1186/1471-2350-12-150)
Supplement: Additional file 4 — Epistatic interactions with the MTHFR rs1801133 SNP and plasma homocysteine. The most statistically significant associations (FDR-adjusted Likelihood Ratio Test P ≤ 0.2) for SNP by MTHFR rs1801133 interactions in relation to the plasma homocysteine phenotype for men in the Normative Aging Study. [file 1471-2350-12-150-S4.DOC]

# Folate network genetic variation, plasma homocysteine, and global genomic methylation content: a genetic association study

Susan M Wernimont^1^, Andrew G Clark^2^, Patrick J Stover^1^, Martin T Wells^3^, Augusto A Litonjua^4^, Scott T Weiss^4^, J Michael Gaziano^5^, Katherine L Tucker^6^, Andrea Baccarelli^7,8^, Joel Schwartz^7^, Valentina Bollati^8^, and Patricia A Cassano^9§^

^1^Division of Nutritional Sciences, Cornell University, Ithaca, NY, USA

^2^Department of Molecular Biology & Genetics, Cornell University, Ithaca, NY, USA

^3^Department of Biological Statistics & Computational Biology, Cornell, Ithaca, NY, USA

^4^Channing Laboratory, Brigham and Women’s Hospital, and Harvard Medical School, Boston, MA, USA

^5^Division of Aging, Brigham & Women's Hospital, Boston, MA, USA

^6^Department of Health Sciences, Northeastern University, Boston, MA, USA

^7^Departments of Environmental Health and Epidemiology, Harvard University, Boston, MA, USA

^8^Center of Molecular and Genetic Epidemiology, Department of Environmental and Occupational Health, Università degli Studi di Milano and IRCCS Fondazione Ca’ Granda Ospedale Maggiore Policlinico, Milan, Italy

^9^209 Savage Hall, Division of Nutritional Sciences, Cornell University, Ithaca, NY, USA

^§^Corresponding author

Email addresses:

SMW: [smw38@cornell.edu](mailto:smw38@cornell.edu)

AGC: [ac347@cornell.edu](mailto:ac347@cornell.edu)

PJS: [pjs13@cornell.edu](mailto:pjs13@cornell.edu)

MTW: [mtw1@cornell.edu](mailto:mtw1@cornell.edu)

AAL: [ALITONJUA@PARTNERS.ORG](mailto:ALITONJUA@PARTNERS.ORG)

STW: [scott.weiss@channing.harvard.edu](mailto:scott.weiss@channing.harvard.edu)

JMG: [jmgaziano@partners.org](mailto:jmgaziano@partners.org)

KLT: [KL.Tucker@neu.edu](mailto:KL.Tucker@neu.edu)

AB: [abaccare@hsph.harvard.edu](mailto:abaccare@hsph.harvard.edu)

JS: [JSCHWRTZ@hsph.harvard.edu](mailto:JSCHWRTZ@hsph.harvard.edu)

VB: [abaccare@hsph.harvard.edu](mailto:abaccare@hsph.harvard.edu)

PAC: [pac6@cornell.edu](mailto:pac6@cornell.edu)

**Additional file 4** Epistatic interactions with the *MTHFR* rs1801133 SNP in relation to plasma homocysteine. The most statistically significant associations (FDR-adjusted P ≤ 0.2) for SNP by *MTHFR* rs1801133 interactions in relation to the plasma homocysteine phenotype for men in the Normative Aging Study^a,b^

| **Gene** | **SNP rs#** | **Coded Genotype** | **Genetic Model**^e^ | **Regression**  **Coefficient for**^f^**:** | **β Coeff.** | **Nominal P_interaction_** | **FDR-adjusted P_interaction_** |
| --- | --- | --- | --- | --- | --- | --- | --- |
| *ALDH1L1* | rs2305230^c,d^ | *AA* | R |  |  |  |  |
|  |  |  |  | M.E. of SNP | -0.14 |  |  |
|  |  |  |  | M.E. of *MTHFR* *CT*^g^ | -0.02 |  |  |
|  |  |  |  | M.E. of *MTHFR* *TT*^g^ | 0.07 |  |  |
|  |  |  |  | SNP by *MTHFR* *CT* Interact. | 0.52 |  |  |
|  |  |  |  | SNP by *MTHFR* *TT* Interact. | **h | 3.89 E-04 | 0.114 |
| *ALDH1L1* | rs11715574^c^ | *CC* | R |  |  |  |  |
|  |  |  |  | M.E. of SNP | -0.14 |  |  |
|  |  |  |  | M.E. of *MTHFR* *CT*^g^ | -0.02 |  |  |
|  |  |  |  | M.E. of *MTHFR* *TT*^g^ | 0.07 |  |  |
|  |  |  |  | SNP by *MTHFR* *CT* Interact. | 0.48 |  |  |
|  |  |  |  | SNP by *MTHFR* *TT* Interact. | **h | 7.29 E-04 | 0.114 |
| *ALDH1L1* | rs3772414^c^ | *GG* | R |  |  |  |  |
|  |  |  |  | M.E. of SNP | -0.14 |  |  |
|  |  |  |  | M.E. of *MTHFR* *CT*^g^ | -0.02 |  |  |
|  |  |  |  | M.E. of *MTHFR* *TT*^g^ | 0.08 |  |  |
|  |  |  |  | SNP by *MTHFR* *CT* Interact. | 0.48 |  |  |
|  |  |  |  | SNP by *MTHFR* *TT* Interact. | -0.22 | 1.11 E-03 | 0.115 |
| *DNMT3A* | rs6733868 | *CC + CG* | D |  |  |  |  |
|  |  |  |  | M.E. of SNP | -0.10 |  |  |
|  |  |  |  | M.E. of *MTHFR* *CT*^g^ | -0.10 |  |  |
|  |  |  |  | M.E. of *MTHFR* *TT*^g^ | -0.11 |  |  |
|  |  |  |  | SNP by *MTHFR* *CT* Interact. | 0.12 |  |  |
|  |  |  |  | SNP by *MTHFR* *TT* Interact. | 0.26 | 1.75 E-03 | 0.137 |

^a^Model adjusted for age and smoking; forward strand allele shown.

^b^No SNPs map to more than one gene.

^c^Sparse data (fewer than 5 individuals per category) for some genotype combinations.

^d^Lower quality SNP.

^e^D:Dominant; R:Recessive; A:Additive; O:Overdominant.

^f^M.E.: Main effect.

^g^Reference group is *MTHFR* rs1801133 *CC.*

^h^Unestimable.
