# Supplementary material for: Folate network genetic variation, plasma homocysteine, and global genomic methylation content: a genetic association study
Source: BMC Med Genet. 2011 Nov 21;12:150. doi: 10.1186/1471-2350-12-150 (PMC3266217; doi:10.1186/1471-2350-12-150)
Supplement: Additional file 5 — Gene-nutrient interactions and plasma homocysteine. The most statistically significant associations (P ≤ 0.02) for SNP by nutrient interactions in relation to the plasma homocysteine phenotype for men in the Normative Aging Study. [file 1471-2350-12-150-S5.DOC]

# Folate network genetic variation, plasma homocysteine, and global genomic methylation content: a genetic association study

Susan M Wernimont^1^, Andrew G Clark^2^, Patrick J Stover^1^, Martin T Wells^3^, Augusto A Litonjua^4^, Scott T Weiss^4^, J Michael Gaziano^5^, Katherine L Tucker^6^, Andrea Baccarelli^7,8^, Joel Schwartz^7^, Valentina Bollati^8^, and Patricia A Cassano^9§^

^1^Division of Nutritional Sciences, Cornell University, Ithaca, NY, USA

^2^Department of Molecular Biology & Genetics, Cornell University, Ithaca, NY, USA

^3^Department of Biological Statistics & Computational Biology, Cornell, Ithaca, NY, USA

^4^Channing Laboratory, Brigham and Women’s Hospital, and Harvard Medical School, Boston, MA, USA

^5^Division of Aging, Brigham & Women's Hospital, Boston, MA, USA

^6^Department of Health Sciences, Northeastern University, Boston, MA, USA

^7^Departments of Environmental Health and Epidemiology, Harvard University, Boston, MA, USA

^8^Center of Molecular and Genetic Epidemiology, Department of Environmental and Occupational Health, Università degli Studi di Milano and IRCCS Fondazione Ca’ Granda Ospedale Maggiore Policlinico, Milan, Italy

^9^209 Savage Hall, Division of Nutritional Sciences, Cornell University, Ithaca, NY, USA

^§^Corresponding author

Email addresses:

SMW: [smw38@cornell.edu](mailto:smw38@cornell.edu)

AGC: [ac347@cornell.edu](mailto:ac347@cornell.edu)

PJS: [pjs13@cornell.edu](mailto:pjs13@cornell.edu)

MTW: [mtw1@cornell.edu](mailto:mtw1@cornell.edu)

AAL: [ALITONJUA@PARTNERS.ORG](mailto:ALITONJUA@PARTNERS.ORG)

STW: [scott.weiss@channing.harvard.edu](mailto:scott.weiss@channing.harvard.edu)

JMG: [jmgaziano@partners.org](mailto:jmgaziano@partners.org)

KLT: [KL.Tucker@neu.edu](mailto:KL.Tucker@neu.edu)

AB: [abaccare@hsph.harvard.edu](mailto:abaccare@hsph.harvard.edu)

JS: [JSCHWRTZ@hsph.harvard.edu](mailto:JSCHWRTZ@hsph.harvard.edu)

VB: [abaccare@hsph.harvard.edu](mailto:abaccare@hsph.harvard.edu)

PAC: [pac6@cornell.edu](mailto:pac6@cornell.edu)

**Additional file 5** Gene-nutrient interactions and plasma homocysteine. The most statistically significant associations (P≤0.02) for SNP by nutrient interactions in relation to the plasma homocysteine phenotype for men in the Normative Aging Study^a-d^

| **Gene** | **rs#** | **Nominal P** | **β Coefficient** | **Chr** | **Coded allele** | **Coded allele frequency (%)** | **Genetic Model**^g^ | **Type**^h^ |
| --- | --- | --- | --- | --- | --- | --- | --- | --- |
| **Folate** |  |  |  |  |  |  |  |  |
| *MTRR* | rs7730643 | 1.87E-03 | -0.38 | 5 | *G* | 19% | R | I |
| *DHFR* | rs1650697^e^ | 2.07E-03 | -0.13 | 5 | *T* | 25% | O | 5’ |
| *DHFR* | rs12517451 | 2.30E-03 | -0.12 | 5 | *A* | 25% | O | 3’ |
| *SARDH* | rs129891 | 2.55E-03 | -0.09 | 9 | *A* | 34% | A | I |
| *DMGDH* | rs532964 | 1.47E-02 | -0.10 | 5 | *T* | 46% | D | CN |
| **Vitamin B-6** |  |  |  |  |  |  |  |  |
| *GGH* | rs4617146 | 1.22E-03 | 0.09 | 8 | *T* | 19% | A | I |
| *GLDC* | rs1929933 | 1.67E-03 | 0.16 | 9 | *G* | 31% | R | I |
| *FTH1* | rs17185413^e^ | 2.16E-03 | 0.20 | 11 | *C* | 23% | R | 3’ |
| *GNMT* | rs11752813 | 4.67E-03 | 0.09 | 6 | *G* | 46% | O | 5’ |
| *MAT1A* | rs1819684 | 9.63E-03 | 0.11 | 10 | *T* | 7% | D | I |
| *SARDH* | rs756682 | 1.22E-02 | 0.08 | 9 | *G* | 36% | D | I |
| *SARDH* | rs2073817 | 1.29E-02 | 0.08 | 9 | *A* | 36% | D | CN |
| *AMT* | rs8897^e^ | 1.34E-02 | 0.08 | 3 | *T* | 27% | D | 5’ |
| *MTHFD1L* | rs7765521^f^ | 1.40E-02 | -0.08 | 6 | *A* | 47% | O | I |
| *SARDH* | rs129891 | 1.70E-02 | 0.05 | 9 | *A* | 34% | A | I |
| *MTHFR* | rs1801133 *CT* | 1.83E-02 | -0.033 | 1 | *T* | 35% | D | CN |
|  | rs1801133 *TT* |  | 0.0053 |  |  |  |  |  |
| **Vitamin B-12** |  |  |  |  |  |  |  |  |
| *MTHFD1L* | rs9478162 | 2.46E-03 | 0.33 | 6 | *A* | 23% | R | I |
| *MTHFD1L* | rs9478908 | 2.89E-03 | 0.32 | 6 | *G* | 25% | R | I |
| *DNMT3A* | rs11678631 | 6.42E-03 | 0.17 | 2 | *A* | 49% | R | I |
| *GGH* | rs4617146 | 7.26E-03 | -0.12 | 8 | *T* | 19% | A | I |
| *BHMT* | rs16876512 | 9.46E-03 | 0.67 | 5 | *T* | 10% | R | 5’ |
| *CTH* | rs663465 | 9.75E-03 | 0.15 | 1 | *G* | 42% | D | 5’ |
| *CTH* | rs648743 | 9.89E-03 | 0.15 | 1 | *C* | 42% | D | 5’ |
| *GNMT* | rs2296805 | 1.66E-02 | 0.19 | 6 | *T* | 41% | R | I |
| *ALDH1L1* | rs3772424 | 1.71E-02 | -0.13 | 3 | *A* | 20% | D | I |
| *GNMT* | rs2296804^e^ | 1.78E-02 | 0.18 | 6 | *G* | 41% | R | I |

^a^Model adjusted for age, smoking, and residuals of plasma folate, plasma vitamin B-6, and plasma vitamin B-12; forward strand allele shown; rs# represents SNP involved in interaction.

^b^No False Discovery Rate-adjusted P values reached significance threshold of 0.2.

^c^No sparse data (fewer than 5 individuals per category) for any genotype categories of these SNPs.

^d^Beta coefficient is for SNP x nutrient interaction

^e^SNP maps to more than one gene (rs1650697 maps to *MSH3;* rs17185413 maps to *BEST1,* rs8897 als maps to *NICN1;* rs2296804 maps to *PEX6*).

^f^Lower quality SNP.

^g^D:Dominant; R:Recessive; A:Additive; O:Overdominant.

^h^5’:5’ region; 3’:3’ region; CN:Coding nonsynonymous; CS:Coding synonymous; I:Intronic.
