# Supplementary material for: Folate network genetic variation, plasma homocysteine, and global genomic methylation content: a genetic association study
Source: BMC Med Genet. 2011 Nov 21;12:150. doi: 10.1186/1471-2350-12-150 (PMC3266217; doi:10.1186/1471-2350-12-150)
Supplement: Additional file 6 — Gene-nutrient interactions and Alu element methylation. The most statistically significant associations (P ≤ 0.02) for SNP by nutrient interactions in relation to the global genomic DNA methylation phenotype (Alu elements) for men in the Normative Aging Study. [file 1471-2350-12-150-S6.DOC]

# Folate network genetic variation, plasma homocysteine, and global genomic methylation content: a genetic association study

Susan M Wernimont^1^, Andrew G Clark^2^, Patrick J Stover^1^, Martin T Wells^3^, Augusto A Litonjua^4^, Scott T Weiss^4^, J Michael Gaziano^5^, Katherine L Tucker^6^, Andrea Baccarelli^7,8^, Joel Schwartz^7^, Valentina Bollati^8^, and Patricia A Cassano^9§^

^1^Division of Nutritional Sciences, Cornell University, Ithaca, NY, USA

^2^Department of Molecular Biology & Genetics, Cornell University, Ithaca, NY, USA

^3^Department of Biological Statistics & Computational Biology, Cornell, Ithaca, NY, USA

^4^Channing Laboratory, Brigham and Women’s Hospital, and Harvard Medical School, Boston, MA, USA

^5^Division of Aging, Brigham & Women's Hospital, Boston, MA, USA

^6^Department of Health Sciences, Northeastern University, Boston, MA, USA

^7^Departments of Environmental Health and Epidemiology, Harvard University, Boston, MA, USA

^8^Center of Molecular and Genetic Epidemiology, Department of Environmental and Occupational Health, Università degli Studi di Milano and IRCCS Fondazione Ca’ Granda Ospedale Maggiore Policlinico, Milan, Italy

^9^209 Savage Hall, Division of Nutritional Sciences, Cornell University, Ithaca, NY, USA

^§^Corresponding author

Email addresses:

SMW: [smw38@cornell.edu](mailto:smw38@cornell.edu)

AGC: [ac347@cornell.edu](mailto:ac347@cornell.edu)

PJS: [pjs13@cornell.edu](mailto:pjs13@cornell.edu)

MTW: [mtw1@cornell.edu](mailto:mtw1@cornell.edu)

AAL: [ALITONJUA@PARTNERS.ORG](mailto:ALITONJUA@PARTNERS.ORG)

STW: [scott.weiss@channing.harvard.edu](mailto:scott.weiss@channing.harvard.edu)

JMG: [jmgaziano@partners.org](mailto:jmgaziano@partners.org)

KLT: [KL.Tucker@neu.edu](mailto:KL.Tucker@neu.edu)

AB: [abaccare@hsph.harvard.edu](mailto:abaccare@hsph.harvard.edu)

JS: [JSCHWRTZ@hsph.harvard.edu](mailto:JSCHWRTZ@hsph.harvard.edu)

VB: [abaccare@hsph.harvard.edu](mailto:abaccare@hsph.harvard.edu)

PAC: [pac6@cornell.edu](mailto:pac6@cornell.edu)

**Additional file 6** Gene-nutrient interactions and Alu element methylation. The most statistically

significant associations (P≤0.02) for SNP by nutrient interactions in relation to the global genomic

**DNA methylation phenotype (Alu elements) for men in the Normative Aging Study^a^**

| **Gene** | **rs#** | **Nominal P** | **β Coefficient** | **Chr** | **Coded allele** | **Coded allele frequency (%)** | **Genetic Model**^f^ | **Type**^g^ |
| --- | --- | --- | --- | --- | --- | --- | --- | --- |
| **Folate** |  |  |  |  |  |  |  |  |
| *SLC25A32* | rs3098243 | 1.58E-03 | 0.47 | 8 | *C* | 43% | O | I |
| *SARDH* | rs2502741^e^ | 2.78E-03 | -0.44 | 9 | *A* | 50% | O | I |
| *ALDH1L1* | rs4646760 | 1.05E-02 | -0.68 | 3 | *G* | 35% | R | I |
| *SLC46A1* | rs17719944^c^ | 1.08E-02 | -2.82 | 17 | *G* | 7% | R | I |
| *GART* | rs8788 | 1.17E-02 | 0.38 | 21 | *C* | 26% | O | CN |
| *GLDC* | rs4629927 | 1.57E-02 | -0.36 | 9 | *G* | 41% | O | I |
| *DNMT3A* | rs7578575 | 1.82E-02 | 0.35 | 2 | *A* | 31% | O | I |
| **Vitamin B-6** |  |  |  |  |  |  |  |  |
| *AMT* | rs1464567 | 5.27E-04^b^ | -0.47 | 3 | *C* | 42% | D | I |
| *AMT* | rs1464566 | 7.97E-04^b^ | -0.45 | 3 | *G* | 42% | D | I |
| *DNMT3B* | rs1883729 | 1.70E-03^b^ | 0.52 | 20 | *A* | 40% | R | I |
| *TCN2* | rs5749131 | 3.19E-03 | -0.39 | 22 | *A* | 42% | D | 5’ |
| *DNMT3B* | rs2424922 | 5.19E-03 | 0.43 | 20 | *C* | 45% | R | CS |
| *DNMT3B* | rs6058891 | 5.19E-03 | 0.43 | 20 | *C* | 45% | R | CS |
| *DNMT3B* | rs2424914 | 5.31E-03 | 0.43 | 20 | *G* | 45% | R | I |
| *BHMT* | rs10037045 | 5.58E-03 | 0.36 | 5 | *T* | 29% | D | I |
| *CELF1* | rs2242081 | 6.69E-03 | 0.27 | 11 | *C* | 46% | A | I |
| *DNMT3B* | rs6058869^e^ | 9.38E-03 | 0.45 | 20 | *T* | 39% | R | 5’ |
| *TCN1* | rs519221 | 1.24E-02 | -0.59 | 11 | *T* | 27% | R | I |
| *TCN1* | rs557564^e^ | 1.24E-02 | -0.59 | 11 | *T* | 27% | R | I |
| *GLDC* | rs1755617 | 1.33E-02 | 0.36 | 9 | *T* | 24% | O | I |
| *TCN1* | rs34528912 | 1.46E-02 | 0.55 | 11 | *T* | 5% | A | CN |
| *AMT* | rs11922013 | 1.50E-02 | 0.25 | 3 | *C* | 30% | A | I |
| *ALDH1L1* | rs1868138 | 1.79E-02 | 0.32 | 3 | *T* | 20% | O | I |
| *SLC19A3* | rs11694828 | 1.91E-02 | -0.31 | 2 | *A* | 45% | O | I |
| **Vitamin B-12** |  |  |  |  |  |  |  |  |
| *FPGS* | rs10106^e^ | 1.28E-02 | 0.44 | 9 | *G* | 40% | O | 3’ |
| *FPGS* | rs4451422 | 1.44E-02 | 0.43 | 9 | *G* | 40% | O | 3’ |
| *TYMS* | rs699517^d^ | 1.48E-02 | 0.75 | 18 | *T* | 32% | R | 3’ |
| *AHCYL2* | rs1665105 | 1.54E-02 | 0.30 | 7 | *T* | 44% | A | 3’ |

^a^Model adjusted for age, smoking, and residuals of plasma folate, plasma vitamin B-6, and plasma vitamin B-12; forward

strand allele shown; rs# represents SNP involved in interaction; beta coefficient is for SNP x nutrient interaction term.

^b^False Discovery Rate-adjusted P values reached significance threshold of 0.2.

^c^Sparse data (fewer than 5 individuals per category) for some genotype categories of this SNP.

^d^SNP maps to more than one gene (rs699517 also maps to *ENOSF1*).

^e^Lower quality SNP.

^f^D:Dominant; R:Recessive; A:Additive; O:Overdominant.

^g^5’:5’ region; 3’:3’ region; CN:Coding nonsynonymous; CS:Coding synonymous; I:Intronic.
