# Supplementary material for: Folate network genetic variation, plasma homocysteine, and global genomic methylation content: a genetic association study
Source: BMC Med Genet. 2011 Nov 21;12:150. doi: 10.1186/1471-2350-12-150 (PMC3266217; doi:10.1186/1471-2350-12-150)
Supplement: Additional file 7 — Gene-nutrient interactions and LINE-1 element methylation. The most statistically significant associations (P ≤ 0.02) for SNP by nutrient interactions in relation to the global genomic DNA methylation phenotype (LINE-1 elements) for men in the Normative Aging Study [file 1471-2350-12-150-S7.DOC]

# Folate network genetic variation, plasma homocysteine, and global genomic methylation content: a genetic association study

Susan M Wernimont^1^, Andrew G Clark^2^, Patrick J Stover^1^, Martin T Wells^3^, Augusto A Litonjua^4^, Scott T Weiss^4^, J Michael Gaziano^5^, Katherine L Tucker^6^, Andrea Baccarelli^7,8^, Joel Schwartz^7^, Valentina Bollati^8^, and Patricia A Cassano^9§^

^1^Division of Nutritional Sciences, Cornell University, Ithaca, NY, USA

^2^Department of Molecular Biology & Genetics, Cornell University, Ithaca, NY, USA

^3^Department of Biological Statistics & Computational Biology, Cornell, Ithaca, NY, USA

^4^Channing Laboratory, Brigham and Women’s Hospital, and Harvard Medical School, Boston, MA, USA

^5^Division of Aging, Brigham & Women's Hospital, Boston, MA, USA

^6^Department of Health Sciences, Northeastern University, Boston, MA, USA

^7^Departments of Environmental Health and Epidemiology, Harvard University, Boston, MA, USA

^8^Center of Molecular and Genetic Epidemiology, Department of Environmental and Occupational Health, Università degli Studi di Milano and IRCCS Fondazione Ca’ Granda Ospedale Maggiore Policlinico, Milan, Italy

^9^209 Savage Hall, Division of Nutritional Sciences, Cornell University, Ithaca, NY, USA

^§^Corresponding author

Email addresses:

SMW: [smw38@cornell.edu](mailto:smw38@cornell.edu)

AGC: [ac347@cornell.edu](mailto:ac347@cornell.edu)

PJS: [pjs13@cornell.edu](mailto:pjs13@cornell.edu)

MTW: [mtw1@cornell.edu](mailto:mtw1@cornell.edu)

AAL: [ALITONJUA@PARTNERS.ORG](mailto:ALITONJUA@PARTNERS.ORG)

STW: [scott.weiss@channing.harvard.edu](mailto:scott.weiss@channing.harvard.edu)

JMG: [jmgaziano@partners.org](mailto:jmgaziano@partners.org)

KLT: [KL.Tucker@neu.edu](mailto:KL.Tucker@neu.edu)

AB: [abaccare@hsph.harvard.edu](mailto:abaccare@hsph.harvard.edu)

JS: [JSCHWRTZ@hsph.harvard.edu](mailto:JSCHWRTZ@hsph.harvard.edu)

VB: [abaccare@hsph.harvard.edu](mailto:abaccare@hsph.harvard.edu)

PAC: [pac6@cornell.edu](mailto:pac6@cornell.edu)

**Additional file 7** Gene-nutrient interactions and LINE-1 element methylation. The most statistically

significant associations (P≤0.02) for SNP by nutrient interactions in relation to the global genomic

DNA methylation phenotype (LINE-1 elements) for men in the Normative Aging Studya,c,e

| **Gene** | **rs#** | **Nominal P** | **β Coefficient** | **Chr** | **Coded allele** | **Coded allele frequency (%)** | **Genetic Model**^f^ | **Type**^g^ |
| --- | --- | --- | --- | --- | --- | --- | --- | --- |
| **Folate** |  |  |  |  |  |  |  |  |
| *FOLH1* | rs664584 | 1.53E-02 | -0.52 | 11 | *A* | 24% | A | I |
| *BHMT* | rs16876512 | 1.55E-02 | -3.55 | 5 | *T* | 10% | R | 5’ |
| *MAT1A* | rs1985908 | 1.56E-02 | 0.62 | 10 | *C* | 32% | D | 3’ |
| *DMGDH* | rs28326 | 1.96E-02 | 0.58 | 5 | *G* | 18% | A | I |
| **Vitamin B-6** |  |  |  |  |  |  |  |  |
| *MTHFD1L* | rs17080689 | 5.40E-05^b^ | -1.10 | 6 | *C* | 11% | O | I |
| *FTH1* | rs1800009^d^ | 3.59E-03 | 0.65 | 11 | *G* | 34% | O | 3’ |
| **Vitamin B-12** |  |  |  |  |  |  |  |  |
| None |  |  |  |  |  |  |  |  |

^a^Model adjusted for age, smoking, and residuals of plasma folate, plasma vitamin B-6, and plasma vitamin B-12; forward strand allele shown; rs# represents SNP involved in interaction; beta coefficient is for SNP x nutrient interaction term.

^b^False Discovery Rate-adjusted P values reached significance threshold of 0.2.

^c^No sparse data (fewer than 5 individuals per category) for any genotype categories of these SNPs.

^d^SNP maps to more than one gene (rs1800009 also maps to *BEST1*).

^e^No lower quality SNPs.

^f^D:Dominant; R:Recessive; A:Additive; O:Overdominant.

^g^5’:5’ region; 3’:3’ region; CN:Coding nonsynonymous; CS:Coding synonymous; I:Intronic.
